# The Amyloid Plaque Proteomes of Alzheimer’s Disease and Mild Cognitive Impairment

**DOI:** 10.21203/rs.3.rs-9012095/v1

**Published:** 2026-03-08

**Authors:** Dominique Leitner, Evgeny Kanshin, Kaleah Balcomb, Manor Askenazi, Jianina Suazo, Mitchell Marta-Ariza, Julie schneider, Beatrix Ueberheide, Eleanor Drummond, Thomas Wisniewski

**Affiliations:** New York University; New York University; The University of Sydney; Biomedical Hosting LLC; New York University; New York University; Rush University; New York University; New York University; New York University

**Keywords:** amyloid, plaques, proteomics, mild cognitive impairment, Alzheimer’s disease

## Abstract

Amyloid plaques contain numerous proteins in addition to amyloid beta in Alzheimer’s disease (AD). Previous localized proteomics identified plaque-associated proteins in late-onset AD, early-onset AD, rapidly progressive AD, preclinical AD, and AD in Down syndrome, although most studies had smaller cohorts and focused primarily on severe pathology. The amyloid plaque proteome in mild cognitive impairment (MCI) has not been evaluated. We evaluated plaque proteomes in MCI and AD with comparisons to neighboring non-plaque tissue and control non-plaque tissue from ROSMAP (151 cases (n = 240 samples); control (n = 62), MCI (n = 36), AD (n = 53)). Tissue was microdissected from autopsy paraffin embedded temporal cortex and evaluated by label-free quantitative proteomics. We identified differentially abundant proteins with robust differences at a false discovery rate (FDR) < 5% and fold-change > 1.5 for 135 proteins in MCI and 156 in AD plaque tissue compared to neighboring non-plaque tissue, which included proteins described previously as well as novel proteins. Gene ontology (GO) term associations included increased inflammatory response and lysosome proteins in both MCI and AD, and decreased myelin proteins particularly in AD. Of plaque proteins altered in at least one disease group, many changed in the same fold-change direction (p < 0.0001, R^2^ = 0.53) and there were 100 shared proteins in MCI and AD. In non-plaque tissue, there were 277 differentially abundant proteins in MCI and 177 in AD; associated with structural constituent of chromatin in MCI and negative regulation of DNA recombination and autolysosome in AD, with decreased proteins associated with actin-myosin filament in AD. Weighted gene correlation network analysis (WGCNA) identified proteins associated with pathology and demographics. We have conducted the most extensive proteomics of microdissected plaque proteomes in both MCI and AD. Our results provide insights into MCI and AD molecular mechanisms, novel biomarkers, and potential novel therapeutic targets.

## Introduction

Amyloid plaque deposition increases in a spatiotemporal pattern in the brain during the progression of Alzheimer’s disease (AD) [62]. In addition to amyloid beta (Aβ), amyloid plaques contain many other proteins that have been identified by various studies, in particular unbiased localized proteomic analyses [20, 19, 93, 4, 56]. Previous proteomic studies identified plaque associated proteins in late-onset AD (LOAD), early-onset AD (EOAD), rapidly progressive AD (rpAD), preclinical AD, and AD in Down syndrome (DS) [20, 19, 93, 56]. These studies showed that amyloid plaques from these different disease subtypes contained a consistent core plaque proteome that was enriched with extracellular matrix, lysosome, and glial proteins. Notably however, the relative enrichment of many of these proteins differed between subtypes. Interestingly, many amyloid plaque enriched proteins have been mechanistically linked to Aβ aggregation or other key pathological features of AD, suggesting that these plaque enriched proteins could have an important role in driving amyloid plaque development or AD progression [19, 57, 20, 5].

Several key questions about the amyloid plaque proteome remain unanswered. It is unclear whether amyloid plaques have the same proteome in those people that are resistant or resilient to cognitive symptom development, those with mild cognitive symptoms, and how this compares to advanced disease. It is critical to determine whether amyloid plaques are biologically dynamic at these different disease stages, as this informs on underlying pathogenesis and can facilitate identification of novel therapeutic strategies. While one prior study characterized the amyloid plaque proteome in preclinical AD defined by the presence of AD pathology without accompanying cognitive impairment, this study was limited by a small sample size of 3 cases [93].

Further, it is unclear how *APOE* genotype influences the amyloid plaque proteome. *APOE* E4 carriers develop amyloid plaques earlier and have an accelerated spread of plaque pathology through the brain [53, 74]. Defining the *APOE* genotype-specific amyloid plaque proteome differences could elucidate the proteins or mechanisms linked to the accelerated pathology development in *APOE* E4 carriers and the protective factors present in *APOE* E2 carriers. It is important to determine whether the amyloid plaque proteome is significantly different due to *APOE* genotype, particularly as it relates to advancing novel proteomic hits towards drug development. A better understanding of these differentially abundant proteins will inform on molecular mechanisms, diagnostic biomarkers, and novel therapeutic strategies.

In this study, we evaluated the plaque proteomes in MCI and AD with comparisons to neighboring non-plaque tissue and control non-plaque tissue. Protein levels were evaluated for associations with case history, including regional pathology levels, *APOE* genotype, and characterized further histologically.

## Methods

### Human brain tissue.

Human brain tissue was obtained under protocols with Institutional Review Board (IRB) approval at NYU Grossman School of Medicine and Rush University. Brain tissue samples come from the Religious Orders Study (ROS) and Memory and Aging Project (MAP) cohorts [7], which had clinical and neuropathology assessment at Rush University [78, 9, 54]. The same initial cohort of control, MCI, and advanced AD cases from our recent CAA study [43] were also evaluated in this study. Regarding clinical criteria, cognitive final consensus diagnosis was performed by a neurologist blinded to post-mortem data (1 = none; 2 = MCI and no other cause; 3 = MCI and another cause; 4 = AD and no other cause). When AD and another cause of cognitive impairment was identified, these cases were excluded. Regarding neuropathology, inclusion criteria was with an ABC score [62] of A0–1/B0–2/C0–1 for control, A2–3/B1–2/C2–3 for MCI, and A3/B3/C3 for AD. Braak staging of tau pathology was also characterized and evaluated in analyses. Cases with high co-pathology of TDP-43 and Lewy bodies were excluded. With these criteria, formalin-fixed paraffin embedded (FFPE) small samples of the inferior temporal cortex were obtained from control (n = 62), MCI (n = 74), and AD cases (n = 56). The 62 control cases included 3 cases with Braak stage 0 and C0, 38 cases with Braak stage 1–4 and C0, and 21 cases Braak stage 1–4 and C1–2 (Supplementary Table 1). A subset of control cases may be considered primary age-related tauopathy (PART) cases [15], with Braak stage 1–4 and C score 0–1 (n = 38). Regional levels of pathology were evaluated in these 192 cases as described below, and those with sufficient pathology for dissection were evaluated by localized proteomics (total of 151 cases: control n = 62, MCI n = 36, AD n = 53). Control cases were included for comparison to non-plaque tissue, as there was not sufficient amyloid plaque pathology for dissection in enough cases to evaluate the plaque proteome in preclinical AD. Case history is summarized in Table 1 and detailed further in Supplementary Table 1.

### Screening immunohistochemistry (IHC).

To evaluate level of regional plaque pathology in the 192 case cohort, one standard glass slide per case was sectioned from FFPE tissue by the NYU Center for Biospecimen Research and Development Core as in our previous study [43]. Briefly, 8 μm sections were deparaffinized and rehydrated through a series of xylenes and ethanol washes, followed by antigen retrieval with 88% formic acid, and 10 mM sodium citrate with 0.05% tween at pH 6. Sections were blocked in 10% normal horse serum for 1 hour, and incubated in a combination of 4G8 (1:1000, BioLegend #800711) and 6E10 (1:1000, BioLegend #803017) primary antibodies targeting Aβ overnight at 4°C. After washing, sections were incubated in biotinylated mouse secondary antibody (1:1000, Vector Laboratories) for 1 hour, followed by avidin-biotin peroxidase (Vector Laboratories) for 1 hour. After washing, sections were incubated with diaminobenzidine (DAB) chromogen solution (ThermoFisher). After additional washes, sections were coverslipped. Regional semiquantitative pathology scores were determined for each case, including plaque pathology (score 0–5), CAA pathology (score 0–5), and frequency of intravascular and perivascular Aβ score (+, ++, +++) which are detailed in Supplementary Table 1.

### Laser capture microdissection (LCM).

FFPE tissue was cut into 8 μm sections onto LCM PET membrane slides [21, 48, 69, 49, 43] by the NYU Experimental Pathology Core. For MCI and AD cases, plaque and neighboring non-plaque tissue were dissected separately into two samples per case. For control cases, only non-plaque tissue was dissected into one sample per case. All tissue sections were subjected to IHC with 4G8 antibody followed by DAB chromogen counterstained with hematoxylin for all cases. Briefly, sections were deparaffinized and rehydrated through a series of xylenes and ethanol washes, followed by incubation in 0.3% H_2_O_2_ for 20 minutes, blocking in 10% normal goat serum for 1 hour, and incubated with 4G8 primary antibody (1:1000, BioLegend #800711) overnight at 4°C. After washing, sections were incubated in biotinylated mouse secondary antibody (1:1000, Vector Laboratories) for 1 hour, followed by avidin-biotin peroxidase (Vector Laboratories) for 1 hour. After washing, sections were incubated with DAB solution (ThermoFisher) for 10 minutes. Finally, sections were counterstained with hematoxylin (Sigma #MHS16) and air dried overnight in a loosely closed container.

**Figure 1:**
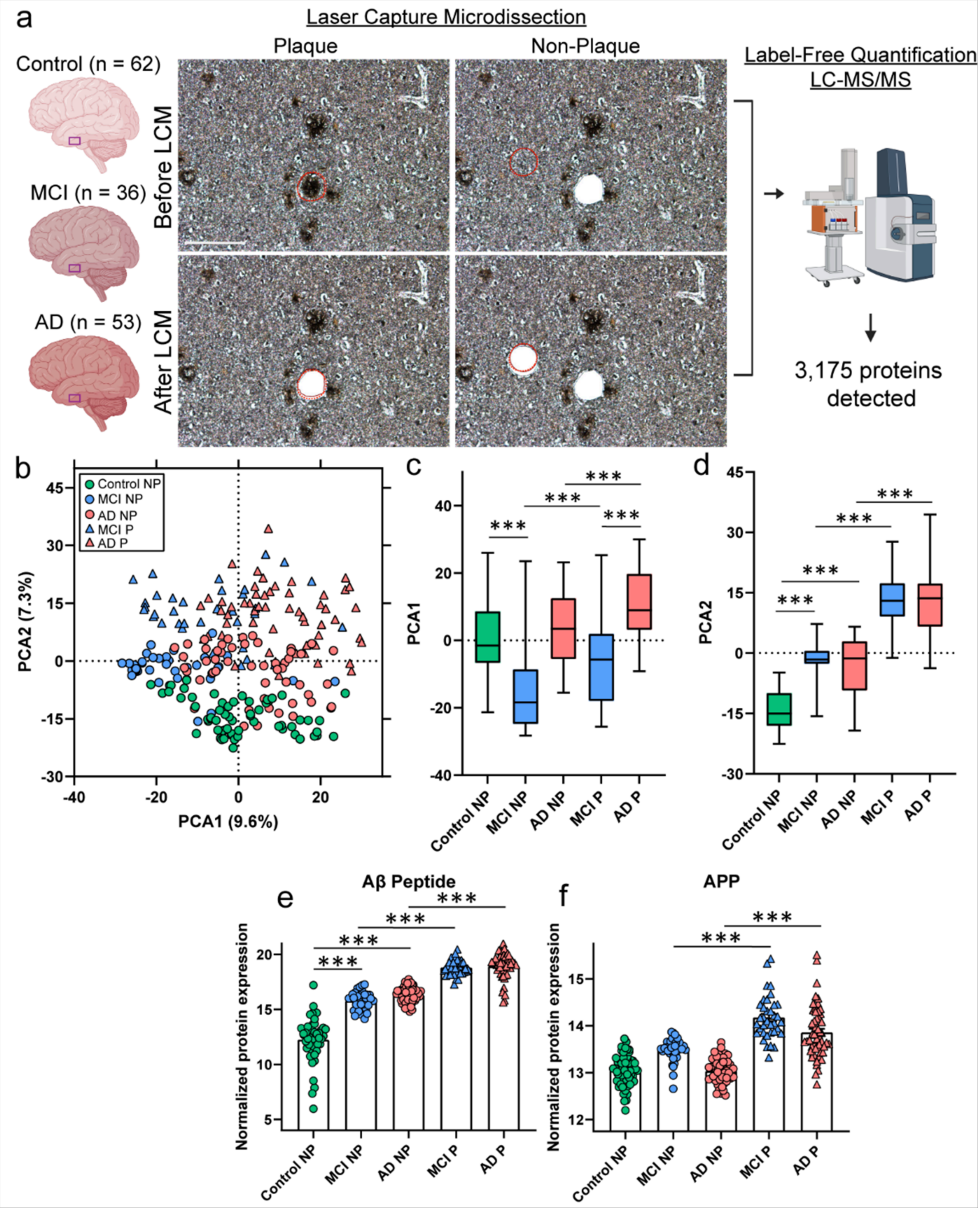
Overview approach for LCM of plaque and neighboring non-plaque tissue. **a)** Plaque tissue and neighboring non-plaque tissue (2 mm^2^) were microdissected by LCM from FFPE human autopsy brain tissue in the inferior temporal cortex from Control (n = 62), MCI (n = 36), and AD (n = 53) cases. Only non-plaque tissue was dissected from control cases, and both plaque and non-plaque tissue was dissected from MCI and AD cases. After dissection, proteins were quantified by label-free quantitative mass spectrometry to identify protein differences, with 3,175 proteins detected. **b)** PCA shows distribution of Control, MCI, and AD plaque and non-plaque tissue samples. **c-d)** The PCA showed significant segregation by disease group and sample type in PCA1 and PCA2, for the pairwise comparisons performed. **e)** The Aβ peptide was enriched in plaque tissue from both MCI and AD, as well as in non-plaque tissue from both MCI and AD when compared to control cases. **f)** APP was enriched in plaque tissue from both MCI and AD. Significant pairwise comparisons are indicated for those analyses that were performed, *** p < 0.001.

Plaque tissue (regardless of plaque morphology and staining intensity, i.e. diffuse and neuritic plaques included) and neighboring non-plaque tissue in the gray matter across all laminar layers of the inferior temporal cortex were dissected at a consistent area per sample of 2 mm^2^ into mass spectrometry (MS) grade water (Thermo Scientific) from n = 62 control (non-plaque tissue), n = 36 MCI (plaque tissue and non-plaque tissue collected separately), AD n = 53 (plaque tissue and non-plaque tissue collected separately). The average area of each plaque was 3,446 um^2^ (standard deviation 500 um^2^) and non-plaque tissue was cut in circles of 3,885 um^2^ (standard deviation 294 um^2^) as depicted in Fig. 1a. To reach 2 mm^2^, this resulted in average plaque number per sample to be 576 (standard deviation 89 plaques, range 403–939). To better match dimensions and technical variables (i.e. laser cut area) and biological variables, non-plaque tissue was cut immediately adjacent to plaque tissue with a set standard size calculated from the average plaque size for the case and a similar size for control non-plaque tissue. Non-plaque tissue included neuropil and cell bodies in the gray matter across all laminar layers of the inferior temporal cortex, with exclusion of blood vessels. Microdissected samples were centrifuged for 2 minutes at 14,000*g* and stored at −80°C. LCM was performed at 10X magnification with a Leica LMD6500 microscope equipped with a UV laser. Schematic overview depicts workflow, partially generated in Biorender (Fig. 1a).

### Label-free quantitative mass spectrometry (LFQ-MS).

Tissue samples were solubilized and digested using the modified SPEED sample prep workflow [18]. Water from LCM samples was removed by vacuum centrifugation and tissue pieces were incubated in 10 μl of formic acid (FA) for 5 minutes at 73°C. FA was then neutralized with 100 μl of 2 M TRIS containing 10 mM TCEP and 20 mM chloroacetamide (CAA), and samples were incubated at 90°C for 60 minutes. For enzymatic digestion, samples were diluted 6x (v:v) with water containing 0.2 μg of sequencing-grade trypsin (Promega) and digested at 37°C overnight with constant shaking at 1,200 rpm. Digestion was halted by acidification to 2% of trifluoroacetic acid (TFA). Peptides were loaded on Evosep Pure C18 tips and analyzed by LC-MS/MS. Online HPLC separation was done on an Evosep One LC system using an 88 min ACN gradient (Evosep extended SPD15 method) on the analytical column (150 μm ID, 15 cm length, #EV-1106). Peptides were identified and quantified in data-independent (DIA) acquisition mode on a QExactive HF-X mass spectrometer (ThermoFisher). High-resolution full MS spectra were acquired with a resolution of 120,000, an AGC target of 3e6, a maximum ion injection time of 60 ms, and a scan range of 350 to 1650 m/z. Following each full MS scan, 22 data-independent HCD MS/MS scans were acquired at the resolution of 30,000, AGC target of 3e6, and stepped normalized collision energy (NCE) of 22.5, 25, and 27.5.

MS raw data were analyzed using Spectronaut software (https://biognosys.com/shop/spectronaut) in directDIA (in-silico generated spectral library) mode against the *Homo* sapiens UniProt reference database (http://www.uniprot.org/) concatenated with a list of common lab contaminants. Database searches were performed in the integrated search engine Pulsar. All precursor and protein IDs were filtered to have FDR < 1%. To allow quantification specifically of the Aβ peptide, instead of the full length APP protein, two separate protein entries were created in the FASTA database: one corresponding to the cleaved toxic Aβ peptide and one representing the rest of the APP sequence. Independent quantification of the Aβ peptide was manually curated and included in search results, as in previous studies [19, 82, 43]. The Aβ peptide identity was verified by inspecting the extracted ions chromatogram traces for both precursor (MS1) and fragment ions (MS2) for peptide LVFFAEDVGSNK (Aβ peptide). This peptide corresponds to amino acids 17–28 of Aβ, and it shows strong enrichment and correlation to Aβ pathology [19, 82, 44, 28, 43, 51]. Protein quantification from the DIA data was performed on the MS2 level using precursor fragment intensities. Protein quantity values were logtransformed and cross-run normalized using median intensity across the samples. Subsequent data analysis was performed in Perseus [87] (http://www.perseus-framework.org/), the R environment (http://www.rproject.org/), and GraphPad Prism v10.4.1. Raw data is available on the MassIVE server (https://massive.ucsd.edu/) under accession MSV000100660.

The protein expression matrix (n = 3,271) was filtered to contain only protein groups that were human and non-lab contaminant (n = 3,175). For principal component analysis (PCA), missing values were imputed from the normal distribution with a width of 0.3 and a downshift of 1.8 (relative to measured protein intensity distribution) in Perseus [87]. PCA1 and PCA2 differences were determined by paired or unpaired t-tests for the groups of interest. Paired t-tests were performed in Perseus v. 1.6.2.3 [87] to detect significant changes in protein abundance between plaque and non-plaque tissue samples in MCI and in AD on non-imputed data. Unpaired t-tests were performed in Perseus to detect significant changes in protein expression between non-plaque samples from control cases compared to MCI or AD non-plaque tissue samples on non-imputed data. Significance was considered at 5% FDR and was further filtered to include proteins with fold-change > 1.5 (Supplementary Table 2). Cell type annotations were derived from a reference data set [41] and as described [47, 69, 45, 49], with 1066 possible annotations from gene IDs that were associated with only one cell type. A comparison of proteins by disease group were evaluated by Venn diagram partially generated from InteractiVenn [30]. Correlation analyses were performed by Pearson correlation. Unsupervised hierarchical clustering and heatmaps were evaluated in the R environment with *ComplexHeatmap* v2.20.0. Individual protein plots depict significance for pairwise comparisons performed and standard error of the mean (SEM).

### Gene ontology (GO) term enrichment analysis.

Differentially abundant proteins identified after pairwise comparisons were evaluated for associated GO biological process (GOBP), cell component (GOCC), and molecular function (GOMF) term enrichment. Canonical isoforms were evaluated, with increased and decreased proteins evaluated separately, against the full proteome as background of the detected proteins (3,145 proteins). Analyses were performed from the UniProt ID with *clusterProfiler* v4.12.0 and *org*.*Hs*.*eg*.*db* v3.19.1 in the R environment v4.4.0. GO terms were considered significant with an adjusted p value < 0.05. The *Simplify* function at 0.7 semantic similarity was used to reduce redundant terms in figures. The top 10 significant simplified terms for each disease group are depicted in figures, and the full list of significant terms are detailed in Supplementary Tables 3–6.

### Protein-protein interaction networks.

Differentially abundant proteins were evaluated by STRING 12.0 and visualized in Cytoscape 3.10.3, with increased and decreased proteins evaluated separately. Protein-protein interaction was evaluated at high confidence (minimum required interaction score = 0.7) and disconnected nodes were hidden.

### IHC characterization.

Follow up histology was performed on HTRA1, CLU, TMEFF2, and RIMS1, with inferior temporal cortex sections from progressive stages of disease [control (n = 12), preclinical AD (n = 10), MCI (n = 12), AD (n = 12)]. For DNAJA1, sections from progressive stages of disease [control (n = 22), preclinical AD (n = 20), MCI (n = 22), AD (n = 23)] were evaluated. Cases were age-matched between groups, and overlapped with cases evaluated by proteomics. All control cases that had a combined plaque and CAA score of ≥2 in IHC screening studies (indicating presence of moderate amyloid pathology in the absence of cognitive impairment) were included in the preclinical AD group. Additional control cases were selected from cases that had a score of 0 for both plaques and CAA in IHC screening studies. Where possible, groups were balanced for sex, age, and *APOE* genotype, detailed in Supplementary Table 1.

Antibodies for CLU (Sigma HPA000572), TMEFF2 (Sigma HPA015587), RIMS1 (Sigma HPA076565), and DNAJA1 (Sigma HPA001306) were selected with consultation from Protein Atlas (www.proteinatlas.org) that has a growing tissue database characterizing antibodies, with the TMEFF2 and DNAJA1 antibodies having an “approved” rating for IHC and the CLU and RIMS1 antibodies having an “enhanced – orthogonal” rating for IHC as antibody staining is mainly consistent with RNA expression across 44 tissues. Further, HTRA1 (R&D Systems MAB29161) antibody targets the same antigen as a similar clone previously described [31], with all 23 detected peptides in our data within the immunogen (Gln23-Pro480). There are three isoforms of TMEFF2 [58], for which the antibody antigen (aa41–175) is contained for all isoforms.

For IHC, FFPE sections (8 μm) were deparaffinized and rehydrated in a series of xylenes and ethanol dilutions. After washing, sections were incubated in 88% formic acid for 7 minutes. Heat-induced antigen retrieval was performed with 10 mM sodium citrate, 0.05% triton-x 100 pH 6. Blocking with 10% normal donkey serum was followed by primary antibodies CLU (1:100) with HTRA1 (1:100) with A17171C-amyloid (1:100, BioLegend #856502), TMEFF2 (1:100) with 4G8 (1:1000), RIMS1 (1:150) with 4G8 (1:1000), and DNAJA1 (1:100) with 4G8 (1:1000) overnight at 4°C. HTRA1 was resuspended in sterile PBS and centrifuged before use to limit precipitates that were not solubilized. Sections were incubated with donkey anti-rabbit 647, donkey anti-mouse 647, donkey anti-mouse 488, or donkey anti-rat 405 (1:500, ThermoFisher), counterstained with DAPI (Sigma D9542) for those slides without donkey anti-rat 405, and coverslipped. Whole slide images were acquired with a Leica Aperio Versa 8 Scanner at 10X magnification with the same settings for each slide for the same protein target. Representative 20X or 40X images were obtained with the Leica Aperio Versa 8 Scanner.

### IHC quantification.

Whole slide images for HTRA1, CLU, TMEFF2, RIMS1, and DNAJA1 were imported into QuPath v0.5.1 [6]. Gray matter was manually annotated to exclude folds, tears, and staining artifact, as well as white matter for RIMS1. A pixel classifier for amyloid (4G8 or A17171C) was then created for quantification of plaques, with size threshold set to exclude below 500 um^2^. Each image was subsequently evaluated and annotations were manually annotated for CAA. The non-plaque area was annotated from a pixel classifier set to below the threshold of the amyloid plaques. Within the amyloid plaque and non-plaque annotations, HTRA1, CLU, TMEFF2, RIMS1, and DNAJA1 were quantified with pixel classifiers. The percent positive area in plaque and non-plaque annotations was calculated for each protein of interest. Intensity was evaluated with defaults by adding intensity features. The same pairwise comparisons were evaluated as those that were performed in the proteomics analyses, with a Wilcoxon matched-pairs or Mann-Whitney to evaluate the paired or group comparisons in GraphPad Prism v10.4.1.

### Comparison with previous studies.

The generated datasets were compared to our NeuroPro version 2.0 database of 55 compiled AD brain tissue proteomic studies [4], with detailed comparison to our previous LOAD plaque dataset [56], our previous MCI and AD CAA proteomic datasets from a subset of the same ROSMAP cases [35], the Global Neurodegeneration Proteomics Consortium (GNPC) biofluid proteomics studies [29], and other recent studies.

The MCI and AD plaque and non-plaque proteomic datasets were compared to the NeuroPro version 2.0 database [4] to identify proteins not described in the 55 studies compiled in this database. NeuroPro contains data from bulk brain tissue, targeted plaque/pTau/CAA lesions, and various soluble fractions from multiple brain regions. Canonical isoforms (Uniprot ID) were evaluated and no other filters were applied. The list of proteins not found in NeuroPro was checked against the gene ID to confirm no known merged IDs were present in the list of proteins not found in NeuroPro.

The MCI and AD plaque proteomic data were compared to our recent plaque proteomic analyses in LOAD, which evaluated more dense plaques in the combined temporal cortex and neighboring hippocampus [56]. Proteins were matched by Uniprot ID and/or gene ID when isoforms detected varied across both studies. Significance was considered as described above for plaque vs non-plaque at 5% FDR and fold change > 1.5, which were also the same thresholds in the previous study. A comparison of proteins across the studies were evaluated by Venn diagram partially generated from InteractiVenn [30]. A Pearson correlation was used to evaluate protein expression levels in plaques across the two studies.

The current plaque datasets (MCI P vs NP, AD P vs NP) were compared to our previous CAA datasets (MCI CAA(+) vs MCI CAA(−), AD CAA(+) vs AD CAA(−)) [43], which included an overlap of the same cases (n = 8 MCI and AD cases). The comparison included proteins detected in both datasets and canonical isoforms, with matching Uniprot ID and/or gene ID (1768 proteins). Significance was considered as described above for plaque vs non-plaque at 5% FDR and fold change > 1.5, and for CAA significance was considered as described previously [43] at p < 0.05 and fold change > 1.5. A comparison of proteins across studies was evaluated by Venn diagram partially generated from InteractiVenn [30]. Two isoforms of MBP were differentially abundant in the current AD plaque dataset, while isoforms were not evaluated in the CAA study and thus both MBP isoforms in the plaque dataset were merged into one Uniprot ID for comparison to CAA and allowed for a comparison of 155 AD plaque proteins to 289 AD CAA proteins. A Pearson correlation was used to evaluate protein expression levels in plaques and CAA.

The current plaque datasets were further evaluated in comparison to 11 proteomic studies that included 2 CAA groups (MCI, AD) [43] and 9 plaque groups from the current dataset of 151 cases (MCI, AD), Marta-Ariza 2025 (DS, EOAD, LOAD) [56], Drummond 2022 (DS, EOAD) [19], and Xiong 2019 (LOAD, preclinical AD) [93]. Proteins were considered significantly enriched in plaques at *p* < 0.05 (t test) and fold change > 1.5 between plaque and non-plaque tissue for [19] and a ratio > 1.5 between plaque and non-plaque tissue for [93]. Remaining proteins that were detected, but not significant between groups were designated as “present”. The significant shared proteins in MCI and AD plaques in the current dataset (100 proteins) were compared against the CAA and other plaque datasets to identify shared and distinct proteins that were enriched in plaque and/or CAA pathology.

The current plaque datasets were compared to the GNPC plasma proteomic data, which was evaluated by Somascan from 16 contributors with AD diagnosis determined clinically by all contributors and diagnosis determined from biological evidence from 11 contributors (AD vs control: n = 1966 AD and n = 5879 control cases; 7289 Somascan aptamers) [33]. From our dataset, the canonical isoform was compared to GNPC proteins. GNPC protein datasets were processed as described and considered significant at Meta_pval_FDR_AD < 0.05 [33]. Proteins were matched by Uniprot ID and/or Entrez gene symbol, with those GNPC proteins that had multiple Uniprot/gene IDs related to different Somascan aptamers compared individually to the current dataset (i.e. NRXN1 had two Uniprot IDs Q9ULB1 and P58400 and there were also different aptamers for the same Uniprot ID for NRXN1).

### Weighted gene correlation network analysis (WGNCA).

Canonical isoforms of proteins (3,145 proteins) were correlated to clinical variables (Supplementary Table 1) by the *WGCNA* package [42] for blockwise modules in the R environment v4.4.0. Disease group and sample type were evaluated separately to better understand the protein differences associated with clinical variables. Defaults were used except where noted. Soft threshold power beta was R^2^ = 0.8, and the soft power for each analysis was: control non-plaque = 5, MCI plaque = 7, MCI non-plaque = 5, AD plaque = 5, AD non-plaque = 4. *APOE* genotype was evaluated as a continuous variable, i.e. *APOE* 22, 23, 33, 34, 44 was coded as 1, 2, 3, 4, 5 respectively to reflect the general association of increasing AD risk similar to other recent studies [95, 89]. GO term enrichment analysis was performed for each protein cluster (module) with the *anRichment* package v1.26 in the R environment v4.4.0 with Entrez IDs against the human GOcollection. GO terms were considered at an FDR < 5%, with the top significant term listed in the figure and all other terms listed in Supplementary Tables 9–13. Proteins associated with regional plaque score in control cases and *APOE* genotype were evaluated further by IHC as described above, RIMS1 and DNAJA1 respectively.

## Results

Proteomic analysis detected 3,175 proteins in plaque and non-plaque tissue from 151 cases (n = 240 total samples) that included control (n = 62), MCI (n = 36), and AD cases (n = 53; Fig. 1a, Supplementary Table 1). PCA showed significant segregation of samples by disease group and sample type in PCA1 and PCA2 (Fig. 1b–d).

Levels of the Aβ peptide and APP in plaque and non-plaque samples were assessed to confirm the successful enrichment of amyloid plaque proteins. In prior studies, the Aβ peptide (LVFFAEDVGSNK) shows a strong enrichment and correlation to Aβ pathology [19, 82, 44, 28, 43, 51], corresponding to amino acids 17–28 of cleaved or full length APP. The LVFFAEDVGSNK Aβ peptide was significantly enriched in plaque tissue from MCI (8.2-fold; p = 1.02 × 10^− 26^) and AD cases (6.2-fold; p = 2.38 × 10^− 28^), as well as in non-plaque tissue of MCI (11.7-fold; p = 5.17 × 10^− 16^) and AD (16.7-fold; p = 3.98 × 10^− 25^) when compared to control cases (Fig. 1e, Supplementary Table 2). APP was significantly enriched in MCI and AD plaque samples (Fig. 1f, Supplementary Table 2).

### Plaque vs Non-Plaque Tissue Differential Abundance

**Figure 2:**
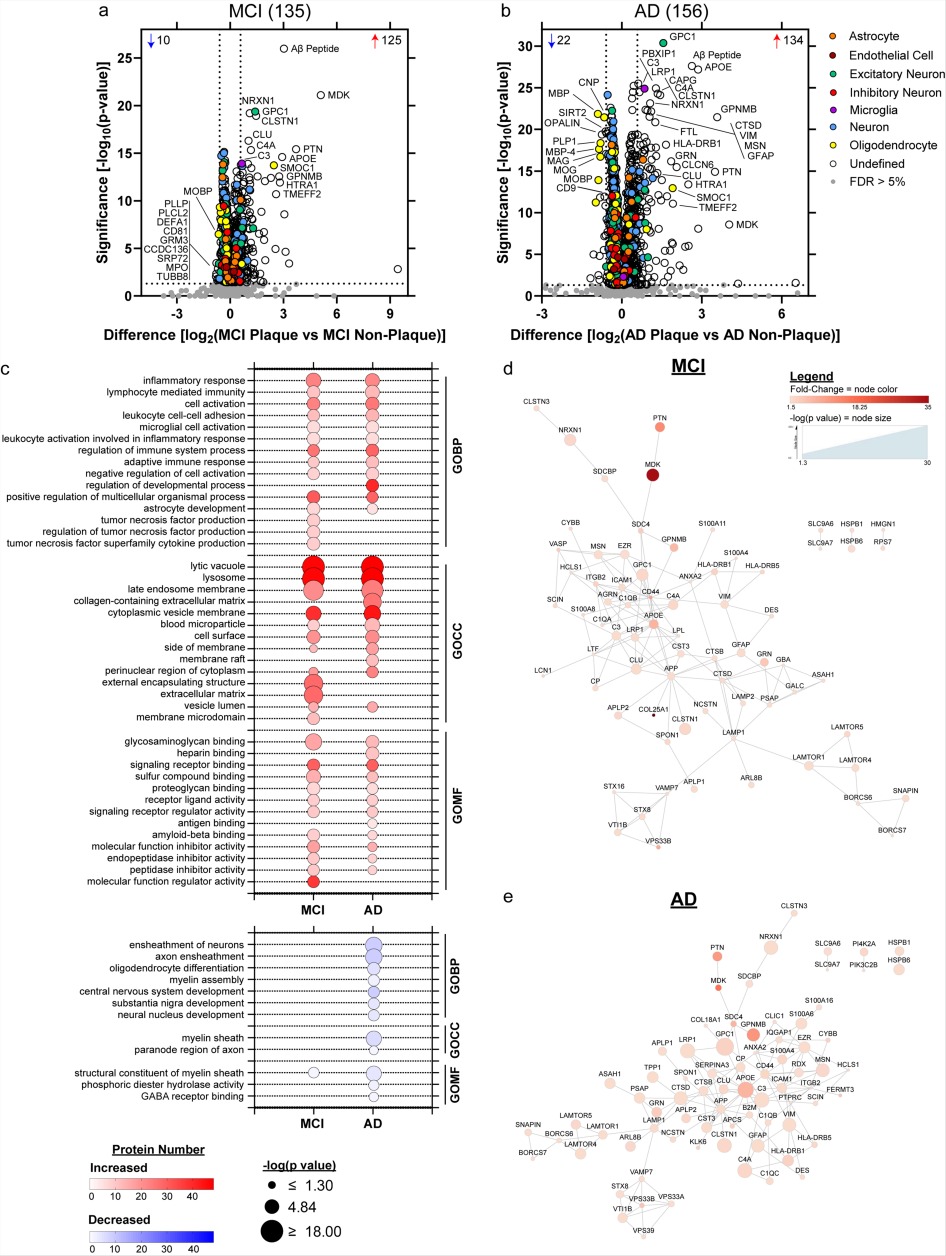
Plaque vs non-plaque tissue differential expression analysis and GO terms in MCI and AD. a) A comparison of MCI plaque vs. MCI non-plaque tissue samples identified 135 differentially abundant proteins (5% FDR, fold-change > 1.5), with 125 increased and 10 decreased. b) A comparison of AD plaque vs. AD non-plaque tissue samples identified 156 differentially abundant proteins, with 134 increased (red) and 22 decreased (blue). The top 10 significantly increased and decreased proteins are annotated by gene name, as well as proteins of interest. Cell type annotations are indicated for each protein. c) Differentially abundant proteins were associated with the indicated top 10 increased (red) or decreased (blue) GO terms in MCI and AD for GO biological process (GOBP), cell component (GOCC), and molecular function (GOMF) terms at adj. p < 0.05 (further detailed in Supplementary Tables 3–4). Terms appear in order of decreasing significance in AD, and corresponding values in MCI are indicated. Number of proteins associated with a term are depicted by color and p value by circle size. d) Increased proteins in MCI plaque tissue were evaluated for high confidence protein-protein interactions, and indicated network enrichment in MCI at p < 1.00 × 10^−16^. Fold-change is indicated by node color, and node size indicates p value. e) Increased proteins in AD plaque tissue were evaluated for high confidence protein-protein interactions, and indicated network enrichment in AD at p < 1.00 × 10^−16^.

Differentially abundant proteins in plaque vs non-plaque tissue were identified in each disease group by paired t-tests at 5% FDR (MCI = 1328 proteins, AD = 1799 proteins, Supplementary Table 2). With fold-change > 1.5 and at 5% FDR, there were 135 proteins differentially abundant in MCI, and 156 proteins in AD (Fig. 2a–b, Supplementary Table 2). The top 10 significant enriched proteins in MCI were Aβ peptide, MDK, GPC1, NRXN1, CLSTN1, CLU, PTN, C4A, APOE, C3 (Supplementary Fig. 1a-i). In AD, the top 10 significant enriched proteins included 7 of these same proteins as well as PBXIP1, CAPG, and LRP1 (while PTN, CLU, MDK were not among the top 10 in AD, they were still significantly enriched; Supplementary Fig. 1). Further, among shared increased proteins, MDK had the highest fold-change in both MCI (34.4-fold; p = 7.86 × 10^− 22^) and AD (16.4-fold; p = 2.69 × 10^− 9^) plaque tissue (detected in 200/240 samples). However, there was some variability of MDK levels within groups, particularly in AD, with a positive correlation in plaque samples to Aβ peptide being mild (p = 0.0026, R^2^ = 0.10) and moderately correlated across all samples (p = 1.56 × 10^− 24^, R^2^ = 0.42). The most significant correlated protein to Aβ peptide in plaque samples was SLC9A7 (p = 3.02 × 10^− 7^, R^2^ = 0.27), and across all samples was APOE (p = 2.45 × 10^− 36^, R^2^ = 0.51) with positive correlations. The most significant correlated protein to Aβ peptide in MCI plaque samples was CLU (p = 3.45 × 10^− 5^, R^2^ = 0.40), and in AD plaque samples was STXBP2 (p = 1.93 × 10^− 7^, R^2^ = 0.45) with positive correlations. Cell type annotations [91] are noted, with the majority of proteins “Undefined” as they are expressed by multiple cell types or it is unknown and thus would require other detailed follow up cell type characterization analysis.

Functional associations with differentially abundant proteins were evaluated next. Top significant GOBP, GOCC, and GOMF terms (FDR adjusted p value < 0.05) were determined for the differentially abundant proteins at 5% FDR and fold-change > 1.5 (Fig. 2c, Supplementary Tables 3–4). The most significant GOBP term associated with increased proteins in both MCI and AD was inflammatory response (MCI: adj. p = 1.65 × 10^− 6^, 23 proteins; AD: adj. p = 1.50 × 10^− 5^, 22 proteins; 18 proteins shared), and with decreased proteins in AD was ensheathment of neurons (AD: adj. p = 1.81 × 10^− 8^, 9 proteins). The most significant GOCC term associated with increased proteins in both MCI and AD was lytic vacuole (MCI: adj. p = 1.03 × 10^− 18^, 47 proteins; AD: adj. p = 1.42 × 10^− 18^, 48 proteins; 40 proteins shared), and with decreased proteins in AD was myelin sheath (AD: adj. p = 7.59 × 10^− 7^, 6 proteins). The most significant GOMF term associated with increased proteins in both MCI and AD was glycosaminoglycan binding (MCI: adj. p = 2.52 × 10^− 8^, 17 proteins; AD: adj. p = 1.45 × 10^− 4^, 13 proteins; 12 shared proteins), and with decreased proteins in both MCI and AD was structural constituent of myelin sheath (MCI: adj. p = 6.87 × 10^− 3^, 2 proteins; AD: adj. p = 1.26 × 10^− 6^, 4 proteins; 2 proteins shared). High confidence protein-protein interactions were determined for the plaque enriched proteins, with network enrichment in MCI at p < 1.00 × 10^− 16^ and in AD at p < 1.00 × 10^− 16^ (Fig. 2d–e).

**Figure 3:**
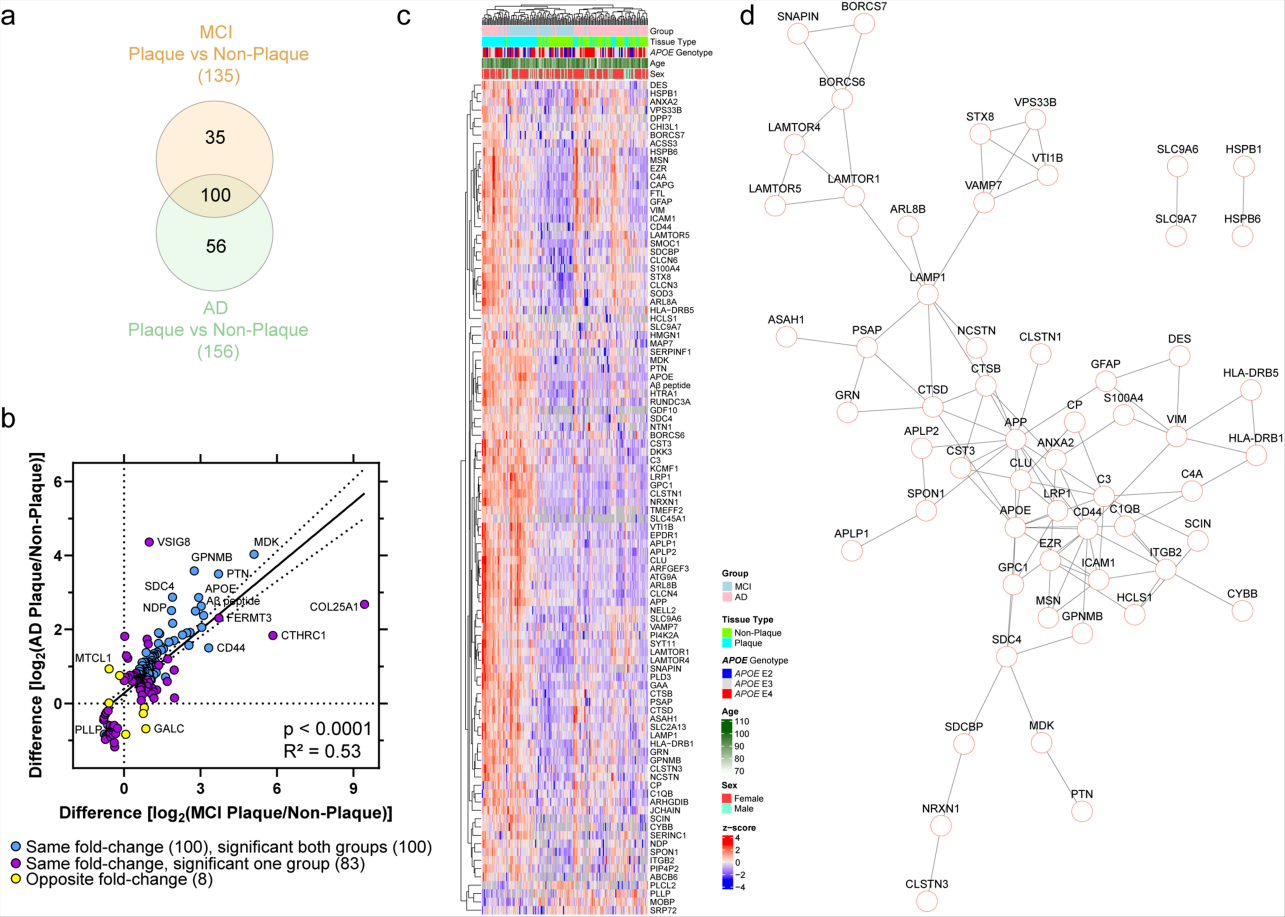
Plaque vs non-plaque tissue protein differences positively correlate in MCI and AD. a) Of the differentially abundant proteins in plaque tissue compared to neighboring non-plaque tissue, there were 191 proteins different in at least one disease group and 100 proteins were shared by both disease groups. Of the 100 shared proteins, all were changing in the same fold-change direction with 96/100 proteins increased in plaque tissue of both disease groups. b) Of the 191 differentially abundant proteins in at least one disease group, there was a positive correlation of protein fold-changes (p < 0.0001, R^2^ = 0.53). There were 96% (183/191) of proteins changing in the same direction (purple, blue) and 4% (8/191) proteins changing in the opposite direction (yellow). The 100 shared proteins changing in the same fold-change direction and significant in both disease groups are indicated in blue. Proteins of interest are annotated by gene name. c) The 100 shared proteins in MCI and AD plaque tissue were evaluated by unsupervised hierarchical clustering, and indicated some clustering by disease group and tissue type. Expression levels are depicted by z-score in the heatmap. d) The 96 shared increased proteins in MCI and AD were evaluated for high confidence protein-protein interactions, and indicated network enrichment at p < 1.00 × 10^−16^.

The amyloid plaque proteomes in AD and MCI were very similar. Of the differentially abundant proteins in at least one disease group (191 proteins), there was a moderate positive correlation of fold-changes (p < 0.0001, R^2^ = 0.53) that indicated many proteins were changing similarly in MCI and AD plaque tissue (Fig. 3a–b). There were 96% (183/191) of proteins changing in the same fold-change direction and only 4% (8/191) of proteins changing in the opposite direction. 100/191 proteins were significantly different in both disease groups and were all changing in the same fold-change direction, with 96/100 proteins increased and 4/100 proteins decreased in both disease groups. Of the 35 proteins significant only in MCI and not in AD, 31 proteins were altered in the same direction in AD but did not reach significance. The few proteins changing in the opposite direction in MCI and AD plaque tissue included CCDC136, C1QTNF4, LPL, and GALC. Of the 56 proteins significant only in AD, 52 proteins changed in the same fold-change direction of MCI cases (proteins with opposite fold-change in SUPT16H, FBP2, MTCL1, IL4I1). Of the 100 shared proteins in MCI and AD, unsupervised hierarchical clustering showed some clustering by disease group and tissue type (Fig. 3c). Among the 96 shared proteins that were significantly enriched in amyloid plaque tissue, the high confidence protein-protein interaction showed network enrichment at p < 1.00 × 10^− 16^ (Fig. 3d). Among shared decreased proteins, these 4 proteins included PLLP, SRP72, MOBP, and PLCL2.

### Non-Plaque Tissue Differential Abundance

**Figure 4:**
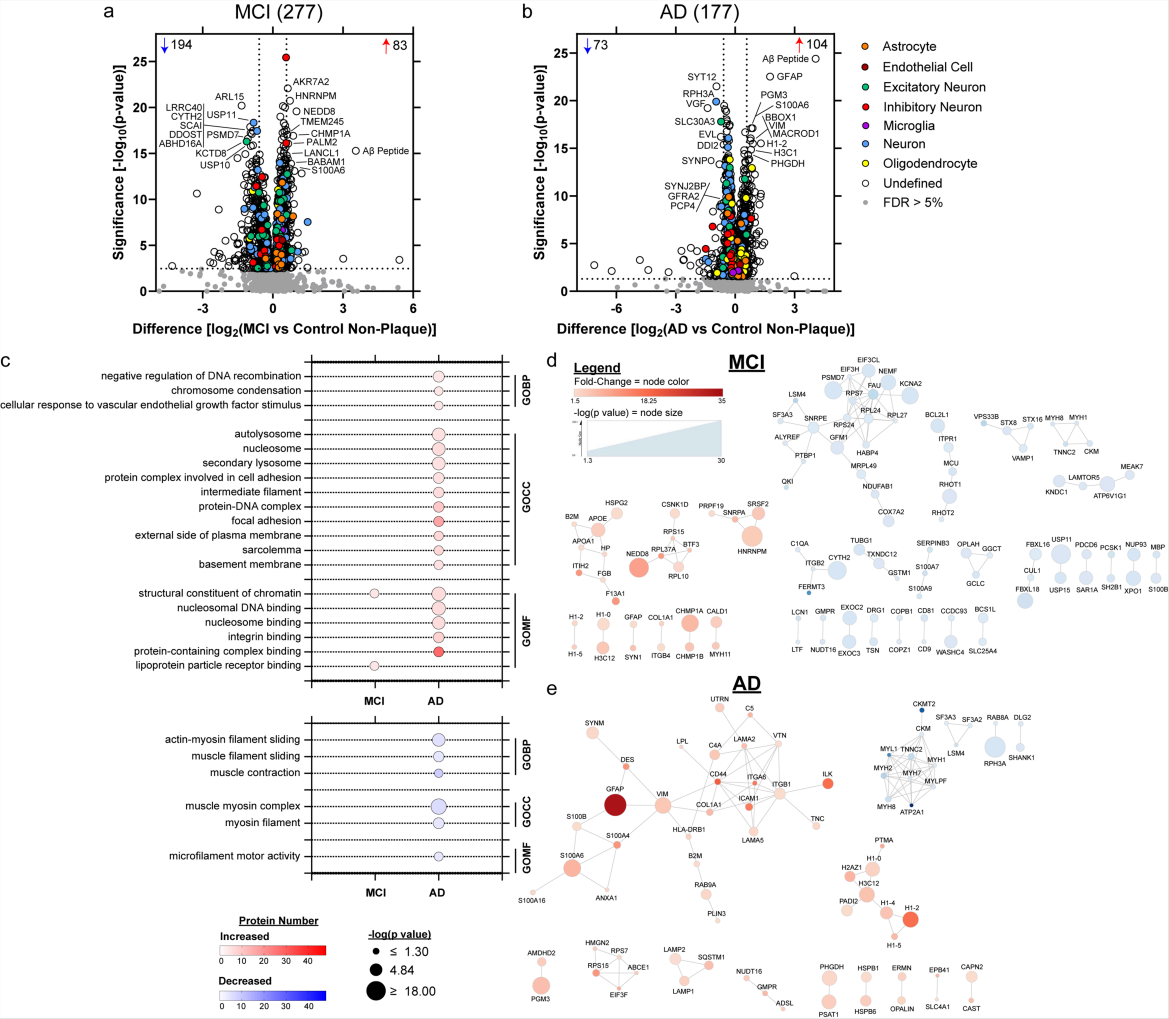
Non-plaque tissue differential expression analysis and GO terms in MCI and AD. **a)** A comparison of MCI non-plaque tissue vs control non-plaque tissue identified 277 differentially abundant proteins (5% FDR, fold-change > 1.5), with 83 increased (red) and 194 decreased (blue). The top 10 significantly increased and decreased proteins are annotated by gene name, as well as other proteins of interest. **b)** A comparison of AD non-plaque tissue vs control non-plaque tissue identified 177 differentially abundant proteins, with 104 increased and 73 decreased. Cell type annotations are indicated for each protein. **c)** Differentially abundant proteins were associated with the indicated top 10 increased (red) or decreased (blue) terms in MCI and AD for GO biological process (GOBP), cell component (GOCC), and molecular function (GOMF) terms at adj. p < 0.05 (further detailed in Supplementary Tables 5–6). Terms appear in order of decreasing significance in AD, and corresponding values in MCI are indicated. Number of proteins associated with a term are depicted by color and p value by circle size. **d)** Increased and decreased proteins in MCI non-plaque tissue were evaluated separately for high confidence protein-protein interactions, and indicated network enrichment for increased proteins at p = 0.011 and decreased proteins at p = 0.0015. Fold-change is indicated by node color, and node size indicates p value. **e)** Increased and decreased proteins in AD non-plaque tissue were evaluated separately for high confidence protein-protein interactions, and indicated network enrichment for increased proteins was at p = 2.65 × 10^−12^ and decreased proteins at p < 1.00 × 10^−16^.

Differentially abundant proteins in MCI and AD non-plaque tissue were identified when compared to control non-plaque tissue. Unpaired t-tests at 5% FDR identified 1422 significantly different proteins in MCI and 1536 in AD. With fold-change > 1.5 and at 5% FDR, there were 277 proteins differentially abundant in MCI, and 177 proteins in AD (Fig. 4a–b). Cell type annotations [91] are noted, with the majority of proteins “Undefined” which was similar to plaque-enriched proteins.

Functional associations with differentially abundant proteins in non-plaque tissue were notably different from those in plaque tissue. Top significant GOBP, GOCC, and GOMF terms (FDR adjusted p value < 0.05) were determined for the differentially abundant proteins at 5% FDR and fold-change > 1.5 (Fig. 4c, Supplementary Tables 5–6). The most significant GOBP term associated with increased proteins in AD was negative regulation of DNA recombination (adj. p = 1.08 × 10^− 2^, 4 proteins: histone proteins H1–0, H1–2, H1–4, H1–5), and with decreased proteins in AD was actin-myosin filament sliding (adj. p = 1.53 × 10^− 3^, 5 proteins: MYH2, MYH7, MYH8, MYL1, MYLPF). The most significant GOCC term associated with increased proteins in AD was autolysosome (adj. p = 1.12 × 10^− 3^, 5 proteins: LAMP1, LAMP2, SQSTM1, FTL, HLA-DRB1), and with decreased proteins in AD was muscle myosin complex (adj. p = 1.06 × 10^− 5^, 6 proteins). The most significant GOMF term associated with increased proteins in both MCI and AD was structural constituent of chromatin (MCI: adj. p = 4.23 × 10^− 2^, 4 proteins; AD: adj. p = 2.88 × 10^− 4^, 6 proteins; 4 proteins shared), and with decreased proteins in AD was microfilament motor activity (adj. p = 4.15 × 10^− 2^, 4 proteins). High confidence protein-protein interactions were determined for differentially abundant proteins, with network enrichment in MCI for increased proteins at p = 0.011 and decreased proteins at p = 0.0015 (Fig. 4d). In AD, high confidence protein-protein interaction network enrichment for increased proteins was at p = 2.65 × 10^− 12^ and decreased proteins at p < 1.00 × 10^− 16^ (Fig. 4e).

**Figure 5:**
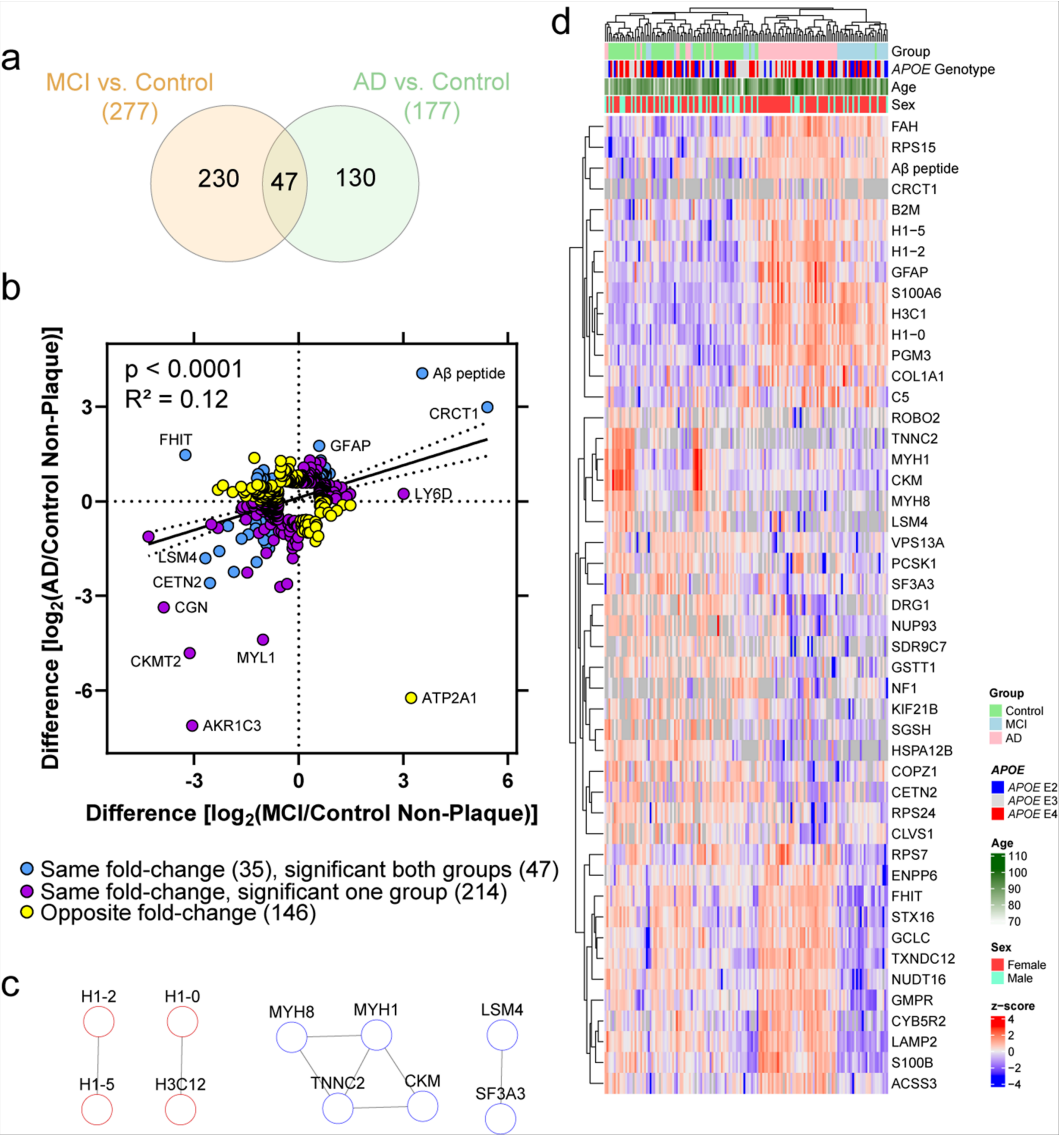
Non-plaque tissue protein differences have a mild positive correlation in MCI and AD. a) Of the differentially abundant proteins in MCI or AD non-plaque tissue when compared to control non-plaque tissue, there were 407 proteins different in at least one disease group and 47 proteins were shared by both disease groups. Of the 47 shared proteins, 21/47 were decreased in non-plaque tissue of both disease groups. b) Of the 407 differentially abundant proteins in at least one disease group, there was a mild positive correlation of protein fold-changes (p < 0.0001, R^2^ = 0.12). There were 61% (249/407) of proteins changing in the same direction (purple, blue) and 39% (158/407) proteins changing in the opposite direction (yellow). The 47 shared proteins significant in both disease groups are indicated in blue, with 35/47 changing in the same fold-change direction and 12/47 in the opposite direction. Proteins of interest are annotated by gene name. c) The 35 shared proteins changing in the same fold-change direction in both MCI and AD were evaluated for high confidence protein-protein interactions, and indicated network enrichment for increased (red nodes) proteins at p = 0.40 and decreased (blue nodes) proteins at p = 2.71 × 10^−4^. d) The 47 shared proteins in MCI and AD in non-plaque tissue were evaluated by unsupervised hierarchical clustering, and indicated some clustering by disease group. Expression levels are depicted by z-score in the heatmap.

Of the differentially abundant proteins in at least one disease group (407 proteins), there was a mild positive correlation of fold-changes (p < 0.0001, R^2^ = 0.12) that indicated that some proteins were changing similarly in MCI and AD non-plaque tissue but with some variability or proteins changing uniquely in a disease group (Fig. 5a–b). There were 61% (249/407) of proteins changing in the same direction and 39% (158/407) of proteins changing in the opposite direction. 47/407 proteins were significantly different in both disease groups and 35/47 proteins were changing in the same fold-change direction with 14/35 shared proteins increased and 21/35 shared proteins decreased. Among the 35 shared proteins changing in the same fold-change direction in both MCI and AD, the high confidence protein-protein interaction for increased proteins showed network enrichment at p = 0.40 and decreased proteins at p = 2.71 × 10^− 4^ (Fig. 5c). Of the 47 shared proteins in MCI and AD, unsupervised hierarchical clustering showed some clustering by disease group (Fig. 5d). Interestingly, among the 47 shared proteins, 12 proteins were significant in both MCI and AD but changing in the opposite fold-change directions with all 12 proteins increased in AD and decreased in MCI (i.e. FHIT, GMPR, CYB5R2, ENPP6, GCLC, NUDT16, STX16, RPS7, ACSS3, LAMP2, TXNDC12, S100B). Among shared increased proteins, the Aβ peptide had the highest fold-change in AD non-plaque tissue (16.7-fold; p = 3.98 × 10^− 25^), and CRCT1 in MCI non-plaque tissue (42.5-fold; p = 3.98 × 10^− 25^) although detected in fewer samples. Among shared decreased proteins, CETN2 had the highest fold-change in AD non-plaque tissue (6.0-fold; p = 4.73 × 10^− 4^), and FHIT had the highest fold-change in MCI non-plaque tissue (9.5-fold; p = 2.37 × 10^− 11^; with opposite fold-change in AD).

### Comparison to Previous Studies

The plaque proteome datasets were compared to our NeuroPro database of 55 compiled AD brain tissue proteomic studies [4], our previous LOAD plaque proteomic dataset [56], our previous CAA proteomic datasets in MCI and AD [43], GNPC biofluid proteomics in AD [33], and other recent studies to validate our findings, better understand protein associations with disease status, and identify novel proteins.

When comparing the current plaque datasets to NeuroPro studies, there were 4 proteins in MCI plaque (all increased: SLC45A1, PIP4P2, PLA2G4B, GBA1), 6 proteins in AD plaque (all increased: IL4I1, VSIG8, SLC45A1, FBP2, PIP4P2, STS), 17 proteins in MCI non-plaque (7 increased, 10 decreased), and 13 proteins in AD non-plaque (2 increased, 11 decreased) that were not included in the NeuroPro v2.0 database and thus are newly reported findings in MCI and AD plaque and non-plaque tissue proteomics (Supplementary Table 7). There was some overlap of these proteins among the different pairwise comparisons, including increased SLC45A1, PIP4P2 in both MCI and AD plaque tissue, and in MCI and AD non-plaque tissue increased CRCT1 and decreased SF3A3, MYHB, TNNC2.

**Figure 6:**
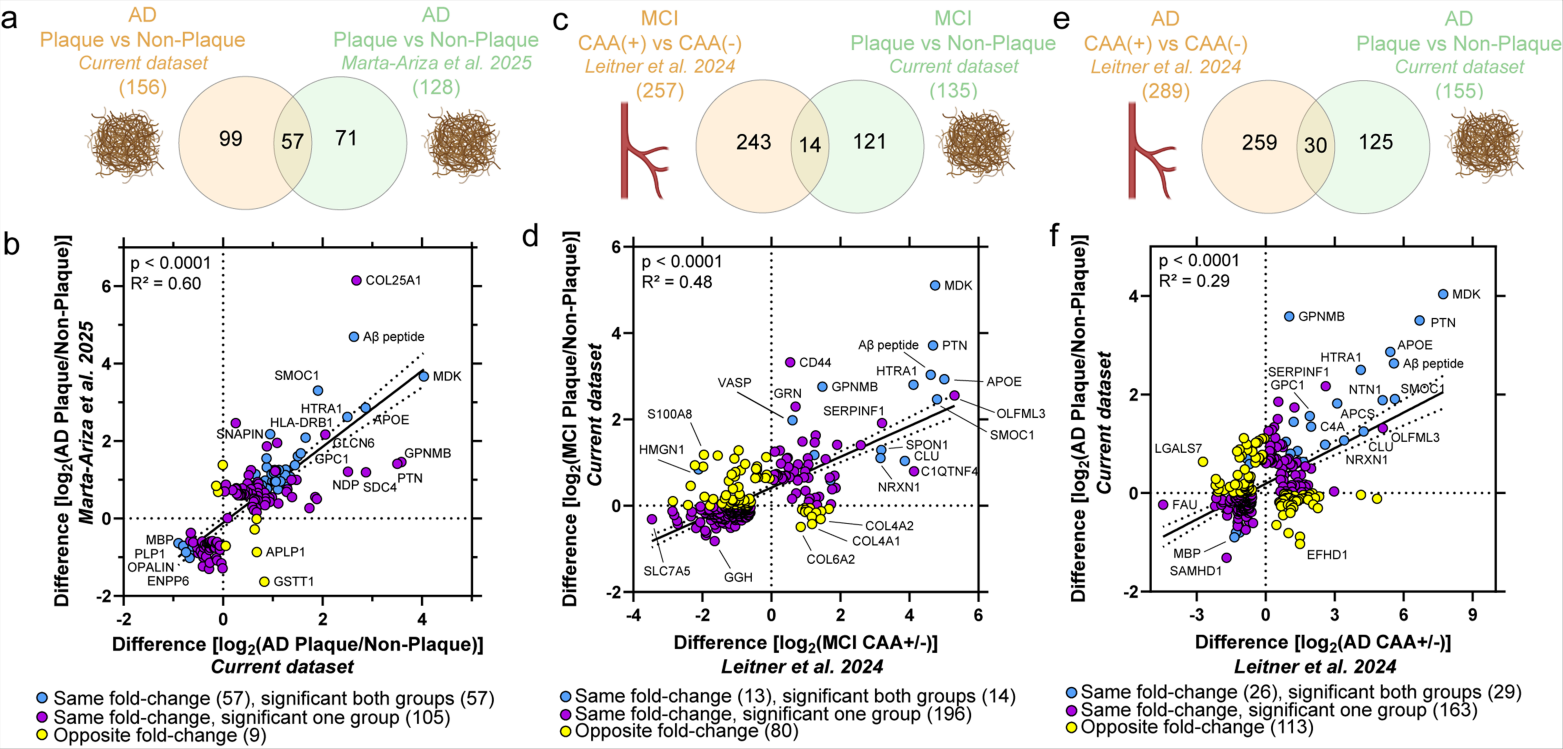
Plaque proteomes vs. other previous proteomic studies. a-b) The AD plaque proteome from the current dataset was compared to our recent LOAD plaque proteomic dataset from Marta-Ariza et al. 2025 [56]. There were 1936 shared detected proteins across both studies. There were 57 differentially abundant proteins shared by both LOAD datasets and all were changing in the same fold-change direction (blue), with 49 increased and 8 decreased proteins. Of the differentially abundant proteins detected and significant in at least one pairwise comparison (171 proteins), there was a moderate positive correlation of protein fold-changes (p < 0.0001, R^2^ = 0.60) that indicated these proteins were changing similarly across studies. c-d) In MCI, there were 290 proteins differentially abundant in at least one pairwise comparison for plaque vs non-plaque from the current dataset or CAA(+) vs CAA(−) in our previous dataset [43]. Of the 290 differentially abundant proteins in at least one group, there was a positive correlation of protein fold-changes (p < 0.0001, R^2^ = 0.48). There were 14 proteins significant in both groups, and 13 proteins were changing in the same fold-change direction (blue). There were 72% (209/290) of proteins changing in the same direction (purple, blue) and 27% (81/290) proteins changing in the opposite direction (yellow). Proteins of interest are annotated by gene name. e-f) In AD, there were 305 proteins differentially abundant in at least one pairwise comparison for plaque vs non-plaque from the current dataset or CAA(+) vs CAA(−) in our previous dataset [43]. The two MBP isoforms differentially abundant in plaques were merged into one Uniprot ID, as isoforms were not evaluated in the CAA study where MBP also differed in CAA samples and allowed for a comparison of 155 AD plaque proteins from the current dataset. Of the 305 differentially abundant proteins in at least one group, there was a positive correlation of protein fold-changes (p < 0.0001, R^2^ = 0.29). There were 29 proteins significant in both groups, and 26 proteins were changing in the same fold-change direction (blue). There were 62% (189/305) of proteins changing in the same direction (purple, blue) and 38% (116/305) proteins changing in the opposite direction (yellow). Proteins of interest are annotated by gene name.

We next compared our plaque datasets directly to one of the NeuroPro studies, our recent localized proteomic study of severe LOAD (A3B3C3 neuropathology) plaque tissue relative to the neighboring non-plaque tissue (1936/1995 detected in the current study; n = 20 cases) [56] that used a similar experimental approach. There were 57/128 (current dataset/previous dataset) differentially abundant proteins shared by both LOAD datasets in the previous and current datasets with 49 increased in both and 8 decreased in both (Fig. 6a). Among shared proteins, the top significant protein with the highest fold-change was MDK. Of the differentially abundant proteins detected and significant in at least one pairwise comparison (171 proteins), there was a moderate positive correlation of fold-changes (p < 0.0001, R^2^ = 0.60) that indicated these proteins were changing similarly across studies (Fig. 6b). Some variation in differentially abundant proteins can be expected and may be associated with brain region evaluated (combined temporal cortex and hippocampus in previous study), exclusion of more diffuse plaques in the previous study, statistical power of the study, and heterogeneity that may come from different cases.

The current MCI and AD plaque datasets were compared directly to one of the NeuroPro studies, our previous CAA proteomic datasets in MCI and AD [43], to identify those proteins enriched similarly or distinctly in amyloid plaques and CAA pathology at each disease stage. This comparison included an overlap of a subset of the same cases (n = 8). Of the differentially abundant proteins detected and significant in at least one pairwise comparison for MCI (290 proteins out of 1768 detected shared proteins), there was a moderate positive correlation in MCI plaque and CAA protein fold-changes (p < 0.0001; R^2^ = 0.48; Fig. 6c–d). Of the differentially abundant proteins detected and significant in at least one pairwise comparison for AD (305 proteins out of 1768 detected shared proteins), there was a mild positive correlation in AD plaque and CAA protein fold-changes (p < 0.0001; R^2^ = 0.29; Fig. 6e–f). Among shared significant proteins in plaque tissue and CAA, MDK was among the proteins with the highest fold-change in both MCI and AD. MDK was at the top for AD and was top in MCI plaques but not in MCI CAA, rather the top protein in MCI CAA was APOE.

**Figure 7:**
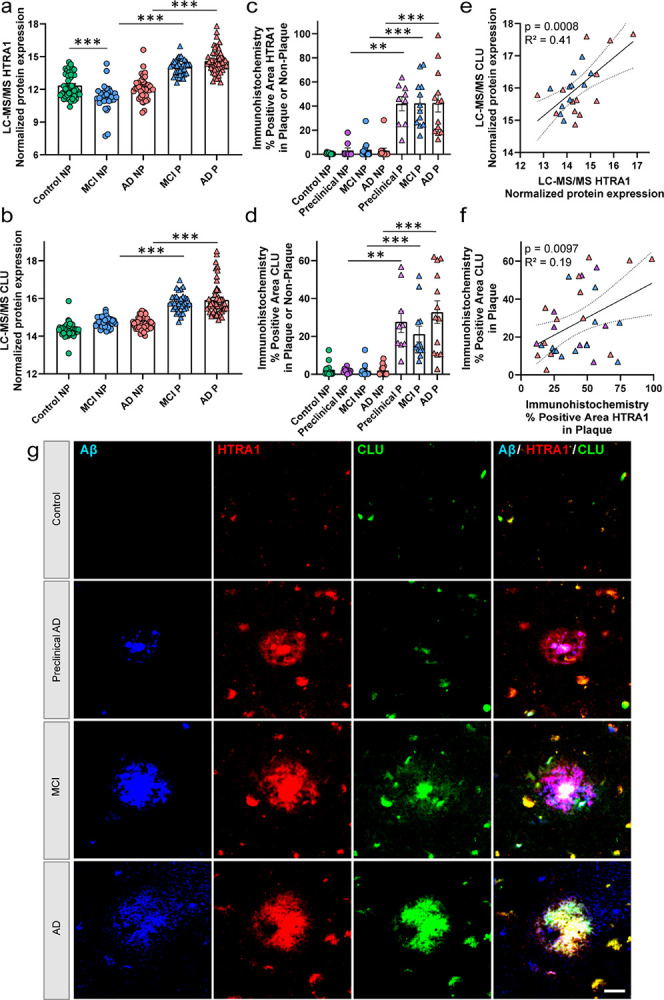
HTRA1 histological characterization. a) Proteomics quantification showed enrichment of HTRA1 in plaque tissue of both MCI (7.0-fold, p = 1.20 × 10^−12^) and AD (5.6-fold, p = 4.08 × 10^−14^), as well as in control vs MCI non-plaque tissue (2.1-fold, p = 4.79 × 10^−5^; detected in 212/240). b) Proteomics quantification of the HTRA1 substrate clusterin (CLU) showed enrichment in plaque tissue of both MCI (2.1-fold, p = 4.97 × 10^−17^) and AD (2.4-fold, p = 2.86 × 10^−15^), with no differences in MCI and AD non-plaque tissue when compared to control non-plaque tissue (detected in 240/240). c) Quantification of HTRA1 immunoreactive area in plaque and non-plaque tissue in temporal cortex sections from progressive stages of disease, Control (n = 12), Preclinical AD (n = 10), MCI (n = 12), and AD (n = 13), showed enrichment for HTRA1 in plaque tissue of preclinical AD, MCI, and AD cases. d) Quantification of CLU immunoreactive area in plaque and non-plaque tissue in temporal cortex sections from progressive stages of disease showed enrichment in plaque tissue of preclinical AD, MCI, and AD cases. e-f) HTRA1 and CLU levels in plaque tissue positively correlated as measured by proteomics (p = 0.0008, R^2^ = 0.41) and immunohistochemistry (p = 0.0097, R^2^ = 0.19). g) Representative images show Aβ, HTRA1, and CLU immunoreactivity at progressive stages of disease. Significant pairwise comparisons are indicated for those analyses that were performed, ** p < 0.01, *** p < 0.001. Scale bar = 20 μm.

The current MCI and AD plaque datasets were next compared directly to several of the NeuroPro studies to further evaluate those proteins enriched similarly or distinctly in amyloid plaques and CAA pathology. This comparison included 2 CAA groups (MCI, AD) from our previous study [43] and 9 plaque groups from the current dataset of 151 cases (MCI, AD), Marta-Ariza et al. 2025 (DS, EOAD, LOAD) [56], Drummond et al. 2022 (DS, EOAD) [19], and Xiong et al. 2019 (LOAD, preclinical AD) [93]. There were 5 proteins enriched across all 11 groups in plaque and CAA pathology: APOE, C4A, HTRA1, SMOC1, and NRXN1. Among these 5 proteins, the largest fold-changes in the MCI and AD plaque tissue from the current dataset were observed for APOE, HTRA1, and SMOC1. We recently further characterized SMOC1 in a subset of the same cases [5]. HTRA1 (High-Temperature Requirement A Serine Peptidase 1) has been previously described in other plaque and CAA studies [93, 19, 97, 31], and we evaluated it here by immunohistochemistry to validate our proteomic findings and further characterize the protein. HTRA1 is a secreted serine protease and plays a role in physiological functions associated with protein quality control with many targets that allow for disaggregation of proteins like APP/Aβ, tubulins, tau, synuclein, TDP-43, FUS [12, 25, 13, 70]. Among the multiple substrates of HTRA1, CLU (clusterin) is a substrate [3] and was detected in our dataset and had a strong correlation to HTRA1 levels across all plaque and non-plaque proteomic samples (p = 2.08 × 10^− 28^, R^2^ = 0.44). Proteomics showed HTRA1 was enriched in plaque tissue in both MCI (7.0-fold, p = 1.20 × 10^− 12^) and AD (5.6-fold, p = 4.08 × 10^− 14^), as well as enriched in control vs MCI non-plaque tissue (2.1-fold, p = 4.79 × 10^− 5^; detected in 212/240 samples; Fig. 7a). Proteomics showed CLU was enriched in plaque tissue in both MCI (2.1-fold, p = 4.97 × 10^− 17^) and AD (2.4-fold, p = 2.86 × 10^− 15^; detected in 240/240 samples, Fig. 7b). By immunohistochemistry, HTRA1 and CLU were enriched in plaque tissue of preclinical AD, MCI, and AD cases (Fig. 7). Further, HTRA1 and CLU levels in plaques positively correlated as measured by proteomics (p = 0.0008, R^2^ = 0.41; Fig. 7e) and immunohistochemistry (p = 0.0097, R^2^ = 0.19; Fig. 7f).

**Figure 8:**
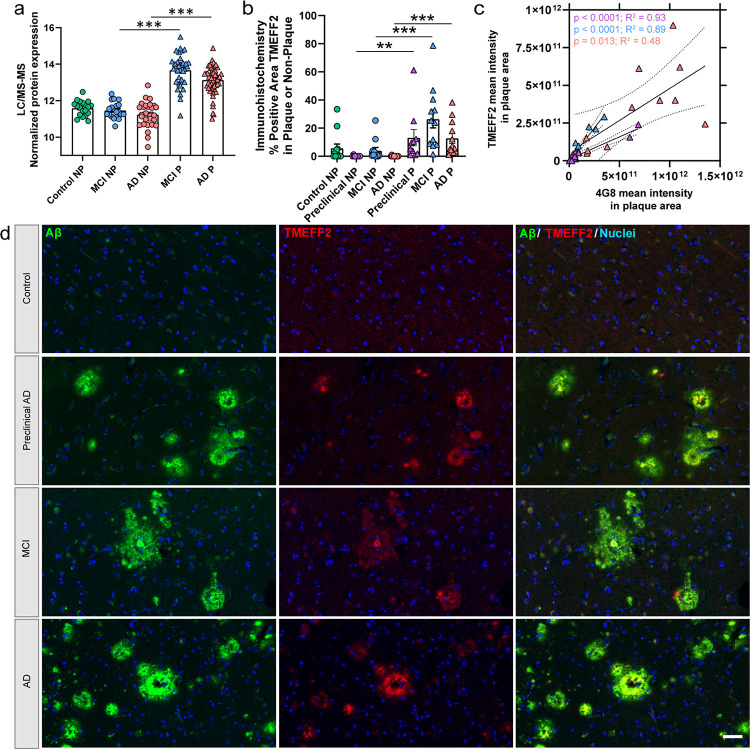
TMEFF2 histological characterization. a) Proteomics quantification showed enrichment of TMEFF2 in plaque tissue in both MCI (6.0-fold, p = 2.02 × 10^−11^) and AD (3.8-fold, p = 8.18 × 10^−12^), with no differences in MCI and AD non-plaque tissue when compared to control non-plaque tissue (detected in 154/240 samples). b) Quantification of TMEFF2 immunoreactive area in plaque and non-plaque tissue in temporal cortex sections from progressive stages of disease, Control (n = 12), Preclinical AD (n = 10), MCI (n = 12), and AD (n = 12), showed enrichment for TMEFF2 in plaque tissue of preclinical AD, MCI, and AD cases. c) TMEFF2 mean intensity from immunohistochemistry correlated to amyloid beta mean intensity in preclinical AD, MCI, and AD, with the strongest correlations in preclinical AD (p < 0.0001, R^2^ = 0.93) and MCI (p < 0.0001, R^2^ = 0.89) and a moderate correlation in AD (p = 0.013, R^2^ = 0.48). d) Representative images show TMEFF2 immunoreactivity at progressive stages of disease. Significant pairwise comparisons are indicated for those analyses that were performed, ** p < 0.01, *** p < 0.001. Scale bar = 50 μm.

We next evaluated these 11 plaque and CAA proteomic datasets to identify proteins that may be distinctly enriched in plaques and low or not present in CAA. Of proteins not detected in our CAA datasets and enriched in ≥ 2 of 3 LOAD plaque groups and were in ≥ 5 of the 9 plaque groups (including both MCI and AD of the current 151 cases), there were 8 proteins (SYT11, SDC4, LAMTOR1, NCSTN, TMEFF2, SCIN, APLP2, SLC9A6). TMEFF2 (tomoregulin 2; also known as transmembrane protein with EGF like and two follistatin domains 2) was enriched in 2 of 3 LOAD groups and 6 of 9 plaque groups overall, with no detection in our CAA study [43] or other CAA studies [97, 67, 31, 34, 28, 55]. Proteomics showed TMEFF2 was enriched in plaque tissue in both MCI (6.0-fold, p = 2.02 × 10^− 11^) and AD (3.8-fold, p = 8.18 × 10^− 12^), detected in fewer control cases with overall detection in 154/240 samples (Fig. 8a). TMEFF2 has previously been described in plaque tissue with no reference to detection in CAA [83], and we evaluated this further by immunohistochemistry in a subset of cases and showed enrichment in plaques from preclinical AD, MCI, and AD cases (Fig. 8b–d). TMEFF2 mean intensity from immunohistochemistry correlated to amyloid beta mean intensity in preclinical AD, MCI, and AD, with the strongest correlations in preclinical AD (p < 0.0001, R^2^ = 0.93) and MCI (p < 0.0001, R^2^ = 0.89) and a moderate correlation in AD (p = 0.013, R^2^ = 0.48). TMEFF2 was also detected on average in a higher percent positive area of plaques in all groups (Fig. 8) than in CAA vessels (Supplementary Fig. 2), where TMEFF2 was detected in perivascular and intravascular regions of a subset of cases and subset of CAA vessels.

The current MCI and AD plaque datasets were compared to GNPC AD plasma proteomic studies [33] to identify plasma proteins that may correspond to brain tissue plaque proteins. This comparison identified 50 shared proteins among all 3 groups (72 proteins shared between MCI and GNPC; 73 proteins shared between AD and GNPC). Of the 50 shared proteins, all were increased in MCI and AD plaque tissue and from GNPC had a positive weighted effect size for 21 proteins with the remainder having a negative weighted effect size (26 proteins) or variable among different Somascan aptamers evaluated (3 proteins; APOE, C4A, LRP1). The top five increased effect sizes from the shared GNPC and plaque proteins came from NRXN1, C3, SMOC1, SPON1, and NTN1 and top five decreased effect sizes were ARL8A, GAA, APP, PSAP, and DES.

### Correlation to Clinical History

**Figure 9:**
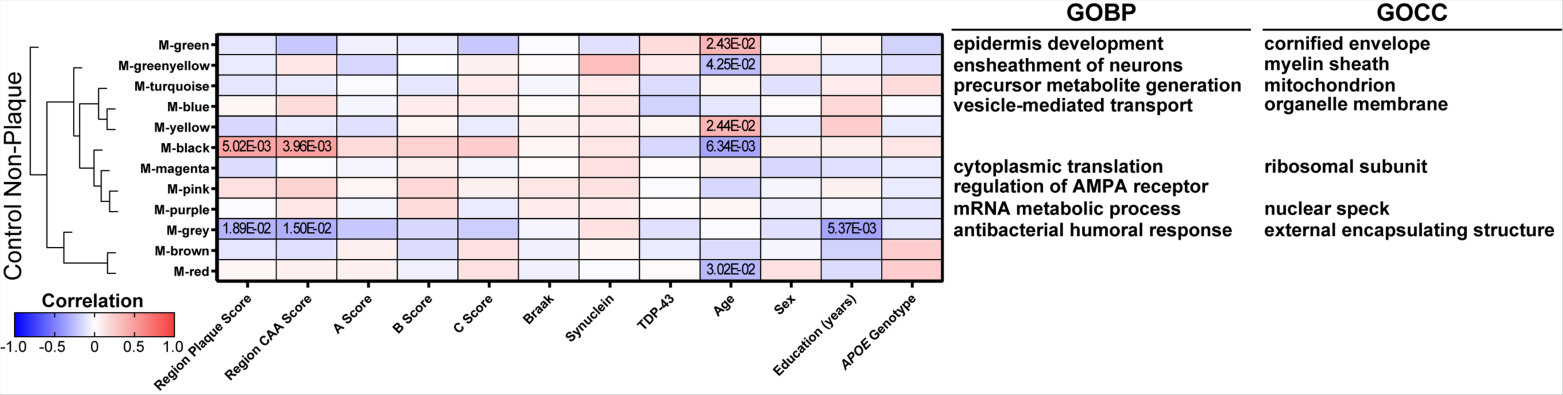
WGCNA of clinical variables in control non-plaque tissue. A correlation analysis of case history variables to proteomics indicated significant protein clusters (“modules”) and associated GO terms in control non-plaque tissue (n = 62). Modules are clustered by eigenprotein adjacency (relatedness to other modules) on the left. Name of module is indicated by “M-color”. P values are indicated for those modules with p < 0.05 correlation. Positive correlation is indicated in red and negative correlation in blue. Top module GOBP and GOCC annotations are noted on the right (FDR<5% with at least 5 proteins) and detailed in Supplementary Table 9.

**Figure 10:**
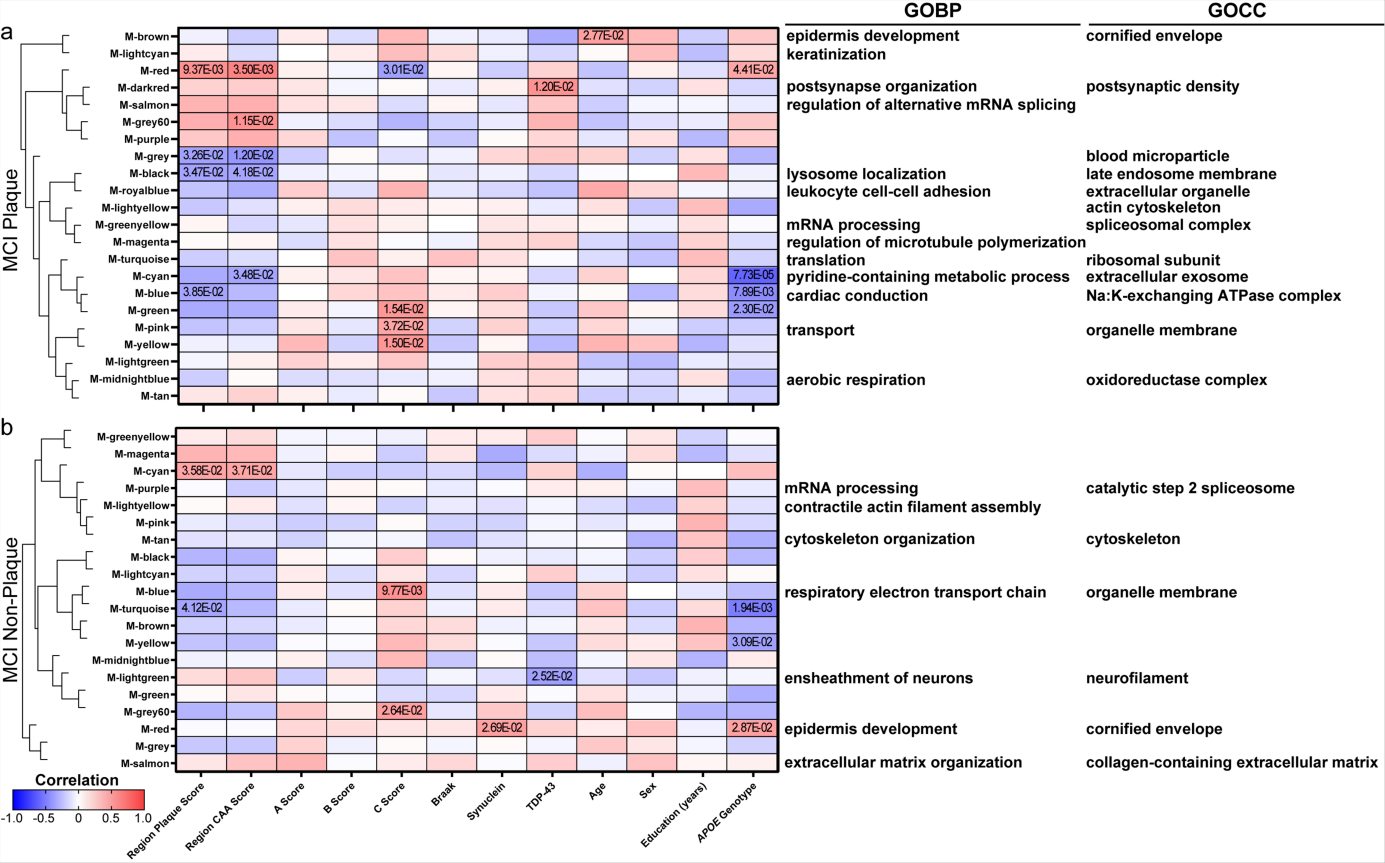
WGCNA of clinical variables in MCI plaque and MCI non-plaque tissue. A correlation analysis of case history variables to proteomics indicated significant protein clusters (“modules”) and associated GO terms in a)MCI plaque tissue and b)MCI non-plaque tissue (n = 36). Modules are clustered by eigenprotein adjacency (relatedness to other modules) on the left. Name of module is indicated by “M-color”. P values are indicated for those modules with p < 0.05 correlation. Positive correlation is indicated in red and negative correlation in blue. Top module GOBP and GOCC annotations are noted on the right (FDR<5% with at least 5 proteins) and detailed in Supplementary Tables 10–11.

**Figure 11:**
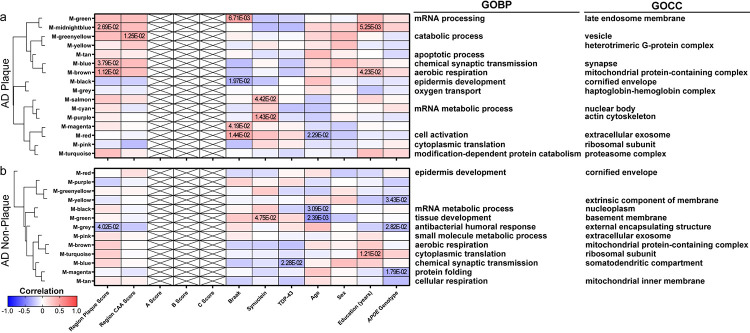
WGCNA of clinical variables in AD plaque and AD non-plaque tissue. A correlation analysis of case history variables to proteomics indicated significant protein clusters (“modules”) and associated GO terms in a) AD plaque tissue and b) AD non-plaque tissue (n = 53). Modules are clustered by eigenprotein adjacency (relatedness to other modules) on the left. Name of module is indicated by “M-color”. P values are indicated for those modules with p < 0.05 correlation. Positive correlation is indicated in red and negative correlation in blue. Top module GOBP and GOCC annotations are noted on the right (FDR<5% with at least 5 proteins) and detailed in Supplementary Tables 12–13.

To identify proteins that correspond to case history, a WGCNA was performed for each disease group and sample type (Figs. 9–11, Supplementary Tables 9–13). There were several protein clusters that corresponded to regional plaque score in all disease groups and sample types, as well as other case history variables.

**Figure 12:**
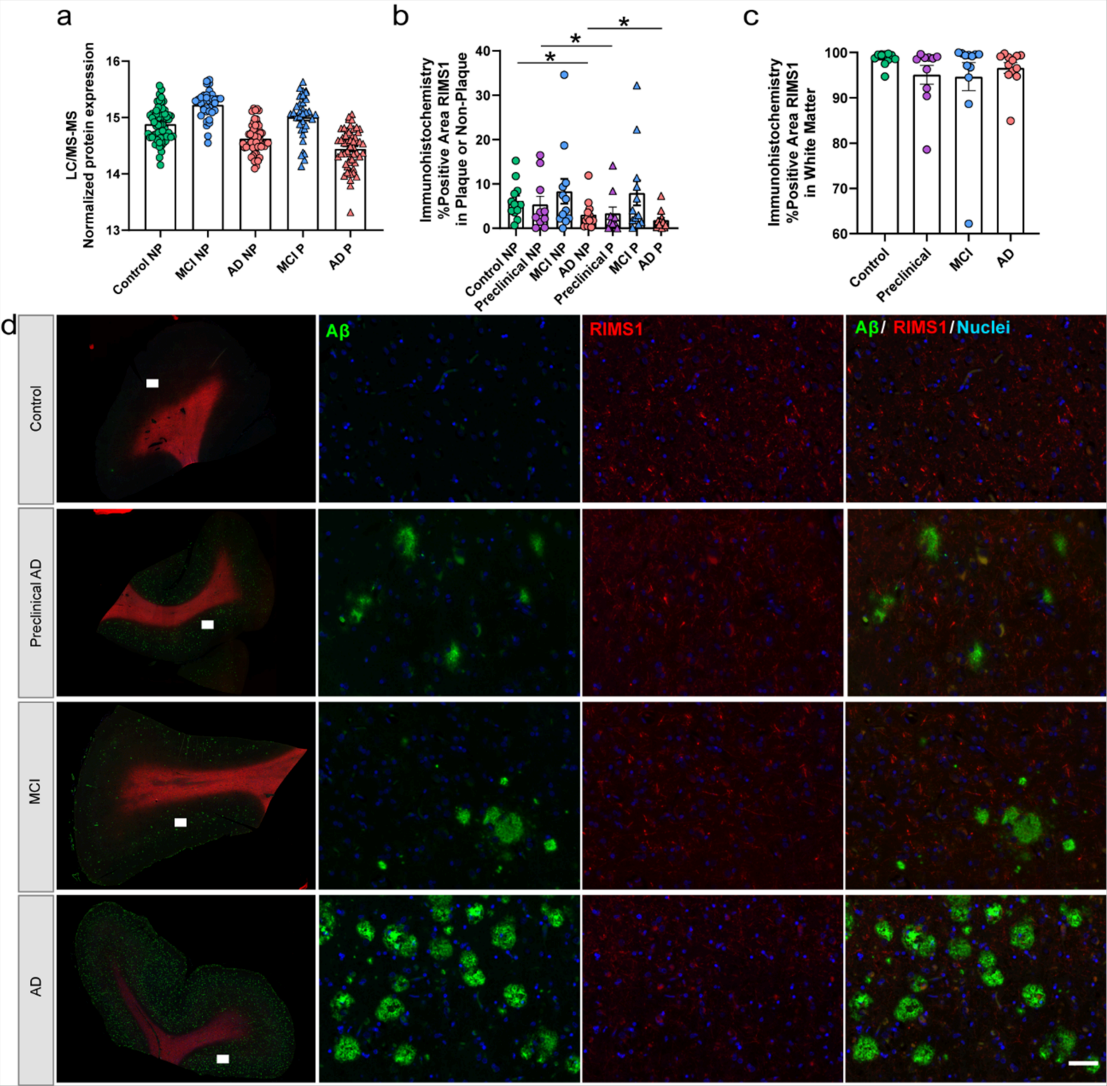
RIMS1 histological characterization. a) In addition to findings by WGCNA where RIMS1 had a positive correlation to regional plaque score in control cases, proteomics quantification of RIMS1 showed differences for all pairwise comparisons performed at FDR < 5% but not at fold-change > 1.5 (detected in 238/240 samples). b) Quantification of RIMS1 immunoreactive area in plaque and non-plaque tissue in temporal cortex sections from progressive stages of disease, Control (n = 12), Preclinical AD (n = 10), MCI (n = 12), and AD (n = 12), showed decreased RIMS1 in AD vs control non-plaque tissue, as well as higher RIMS1 in non-plaque compared to plaque tissue for preclinical AD and AD. c)Histological quantification of RIMS1 in white matter showed no significant difference in preclinical AD, MCI, or AD when compared to control cases. d)Representative images show RIMS1 immunoreactivity in an overview and the indicated inset (white box) at higher magnification at progressive stages of disease. Significant pairwise comparisons are indicated for those analyses that were performed, * p < 0.05, *** p < 0.001. Scale bar = 50 μm.

In control non-plaque tissue (Fig. 9), there were 151 proteins with a positive correlation and 161 proteins with a negative correlation to regional plaque score (n = 39/62 control cases with plaque score ≥ 1), as well as two protein clusters (black, grey) that correlated to regional plaque score. Among the proteins with a positive correlation to plaque score in controls, there was increased RIMS1 (Regulating Synaptic Membrane Exocytosis 1) with increasing plaque score (p = 4.37 × 10^− 4^, R^2^ = 0.19; detected in all samples). In addition to WGCNA, proteomics showed mild differences for RIMS1 in all pairwise comparisons (FDR < 5%, mild FC < 1.5), and included some variability within the groups (MCI P vs NP: 1.16-fold decrease, p = 2.54 × 10^− 3^; AD P vs NP: 1.14-fold decrease, p = 1.13 × 10^− 5^; MCI vs control NP: 1.27-fold increase, p = 6.23 × 10^− 8^; AD vs control NP: 1.19-fold decrease, p = 3.93 × 10^− 6^; Fig. 12a). RIMS1 was evaluated further by immunohistochemistry, which showed decreased RIMS1 in AD vs control non-plaque tissue, as well as higher RIMS1 in preclinical AD and AD non-plaque tissue compared to plaque tissue (Fig. 12b–d). Of note, there was high positivity of RIMS1 in white matter by immunohistochemistry, which did not show a significant difference by disease group (Fig. 12c). The two protein clusters (black = 151 proteins, grey = 442 proteins) that correlated to regional plaque score in control non-plaque tissue included GO terms associated with the grey cluster (top GOBP: antibacterial humoral response; GOCC: external encapsulating structure; Supplementary Table 9) which had a negative correlation to plaque score. The black cluster had a positive correlation to regional plaque score and included RIMS1, but did not have an association with a significant GO term. There were also several clusters that correlated to regional CAA score, age, and years of education in control non-plaque tissue. Those protein clusters with a significant correlation and GO terms included the grey cluster that was negatively correlated to regional CAA score and years of education, in addition to regional plaque score. Other significant protein clusters included the green cluster that positively correlated to age and was related to GOCC term cornified envelope and the greenyellow cluster that negatively correlated to age and was related to GOCC term myelin sheath. There were no protein cluster correlations to other pathology (ABC score, Braak, synuclein, TDP-43), sex, or *APOE* genotype. Regardless of protein cluster correlations, there were 148 proteins that correlated to *APOE* genotype (83 positively correlated; 65 negatively correlated). The most significantly correlated protein to *APOE* genotype was COQ9 (p = 9.10 × 10^− 4^; R^2^ = 0.17) with a negative correlation.

In MCI plaque tissue (Fig. 10a), there were 123 proteins with a positive correlation and 205 proteins with a negative correlation to regional plaque score, as well as four protein clusters (red, grey, black, blue) that correlated to regional plaque score. Those regional plaque protein clusters with a significant correlation and GO terms included negative correlations related to top GOCC terms blood microparticle, late endosome membrane, and Na:K-exchanging ATPase complex (Supplementary Table 10). The top significant protein that correlated to plaque score was a positive correlation to LSM3 (p = 1.08 × 10^− 4^, R^2^ = 0.36), and negative correlation to SERPINC1 (p = 1.38 × 10^− 4^, R^2^ = 0.35). There were also several clusters that correlated to regional CAA score, neuritic plaque C score, TDP-43 pathology, age, and *APOE* genotype. The top significant cluster in MCI plaque tissue and overall in the WGCNA analyses was correlated to *APOE* genotype (cyan; 72 proteins; p = 7.73 × 10^− 5^; R^2^ = 0.37). *APOE* genotype included significant correlations and GO terms for those negatively correlated protein clusters related to top GOCC terms extracellular exosome and Na:K-exchanging ATPase complex (including GAPDH). In addition to protein cluster correlations, there were 406 proteins that correlated to *APOE* genotype (328 negatively correlated, 78 positively correlated). The most significantly correlated protein was DNAJA1 (DnaJ Heat Shock Protein Family (Hsp40) Member A1; p = 1.03 × 10^− 5^; R^2^ = 0.44) with a negative correlation, and was a part of the blue protein cluster. There were no protein cluster correlations to other pathology (plaque A score, tau B score, Braak, synuclein), sex, or years of education.

**Figure 13:**
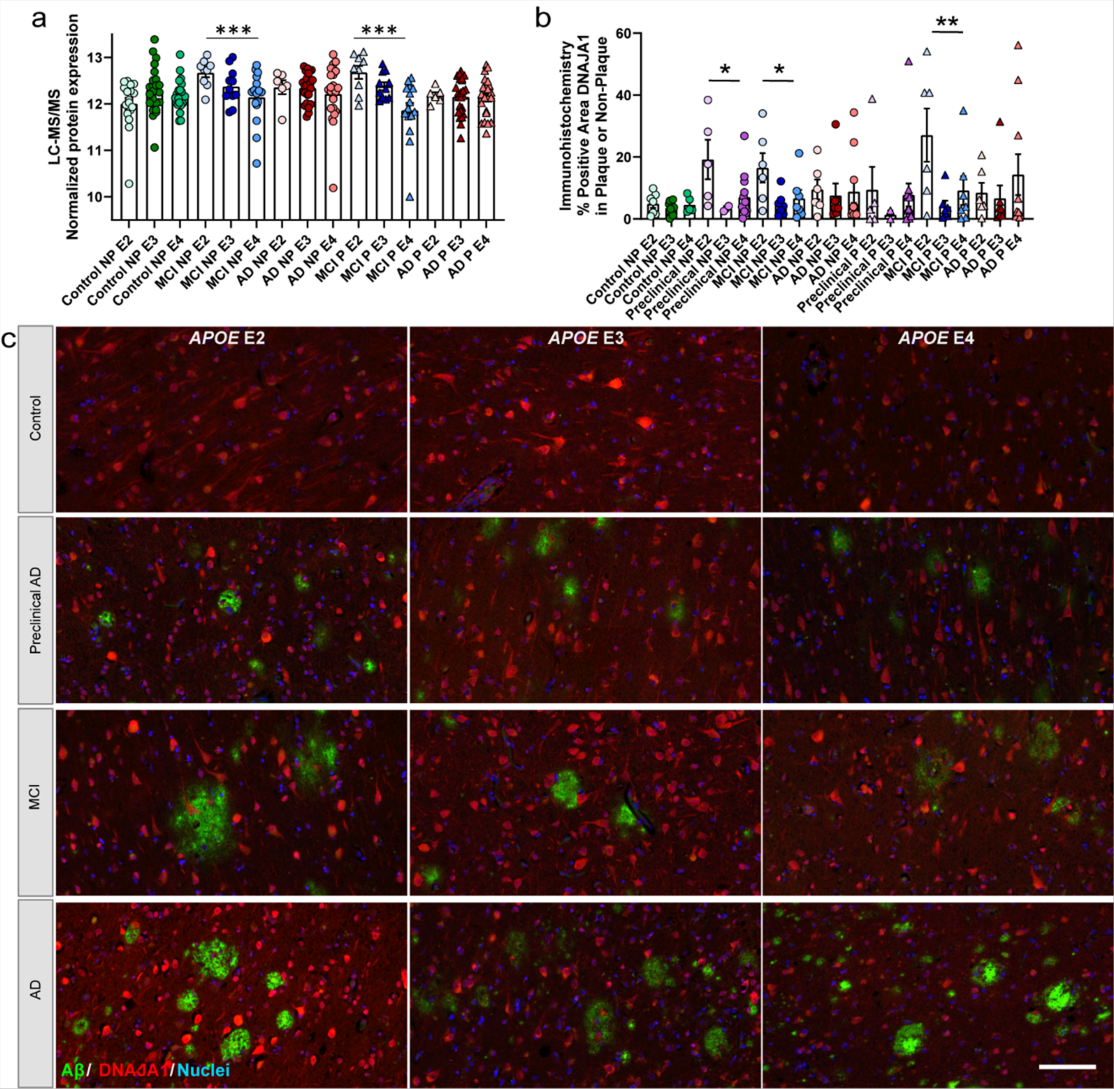
DNAJA1 histological characterization. a)Proteomics quantification of DNAJA1 showed a correlation to *APOE* genotype by WGCNA, particularly in MCI cases of both plaque and non-plaque tissue (detected in 240/240 samples). b) Quantification of DNAJA1 immunoreactive area in plaque and non-plaque tissue in temporal cortex sections from progressive stages of disease, Control (n = 22), Preclinical AD (n = 20), MCI (n = 22), and AD (n = 23), showed a correlation to *APOE* genotype in MCI cases of both plaque and non-plaque tissue as well as in preclinical AD non-plaque tissue by regression analysis with *APOE* genotype as a categorical variable. c)Representative images show DNAJA1 immunoreactivity at progressive stages of disease for each *APOE* genotype group. Significant pairwise comparisons are indicated for those analyses that were performed, * p < 0.05, ** p < 0.01, *** p < 0.001. Scale bar = 100 μm.

In MCI non-plaque tissue (Fig. 10b), there were 116 proteins with a positive correlation and 146 proteins with a negative correlation to regional plaque score, as well as two protein clusters (cyan, turquoise) that correlated to regional plaque score. The regional plaque protein clusters with a significant correlation and GO terms included a negative correlation related to top GOMF term ATP-dependent protein folding chaperone (turquoise cluster; Supplementary Table 11). There were also several clusters that correlated to regional CAA score, neuritic plaque C score, synuclein pathology, TDP-43 pathology, and *APOE* genotype. *APOE* genotype included negative correlation to top GOMF term ATP-dependent protein folding chaperone (turquoise cluster) and positive correlation to top GOCC term cornified envelope (red cluster). In addition to protein cluster correlations, there were 485 proteins that correlated to *APOE* genotype (373 negatively correlated, 112 positively correlated; 210 proteins shared between MCI plaque and non-plaque). The most significantly correlated protein to *APOE* genotype was GAPDH (p = 3.36 × 10^− 5^; R^2^ = 0.40) with a negative correlation. Among the top 20 correlated proteins, DNAJA1 (part of turquoise protein cluster) was also negatively correlated as it was in MCI plaque tissue with higher levels in *APOE* E2 cases and lower levels in *APOE* E3 and *APOE* E4 cases (Fig. 13a). DNAJA1 was evaluated further by immunohistochemistry and showed a correlation to *APOE* genotype in MCI cases of both plaque and non-plaque tissue as well as in preclinical AD non-plaque tissue (Fig. 13b–c). There were no protein cluster correlations to other pathology (plaque A score, tau B score, Braak), age, sex, or years of education.

In AD plaque tissue (Fig. 11a), there were 357 proteins with a positive correlation and 61 proteins with a negative correlation to regional plaque score, as well as three protein clusters (midnightblue, blue, brown) that correlated to regional plaque score. The regional plaque protein clusters with a significant correlation and GO terms included a positive correlation related to top GOCC term mitochondrial protein-containing complex, synapse, and top GOMF term hormone activity (Supplementary Table 12). The top significant protein that correlated to plaque score was a positive correlation to RAB12 (p = 2.95 × 10^− 5^, R^2^ = 0.29), and negative correlation to SERPINC1 (p = 5.32 × 10^− 5^, R^2^ = 0.28). There were also several clusters that correlated to regional CAA score, Braak, synuclein pathology, age, and years of education. There were no protein cluster correlations to TDP-43 pathology, sex, or *APOE* genotype. Regardless of protein cluster correlations, there were 134 proteins that correlated to *APOE* genotype (63 negatively correlated, 71 positively correlated; 13 proteins shared with MCI plaque). The most significantly correlated protein to *APOE* genotype was SET (p = 4.15 × 10^− 5^; R^2^ = 0.28) with a negative correlation.

In AD non-plaque tissue (Fig. 11b), there were 97 proteins with a positive correlation and 65 proteins with a negative correlation to regional plaque score, as well as one protein cluster (grey) that negatively correlated to regional plaque score and was related to top GOCC term external encapsulating structure (Supplementary Table 13). There were also several clusters that correlated to synuclein pathology, TDP-43 pathology, age, years of education, and *APOE* genotype. For *APOE* genotype, there were significant cluster and GO terms with negative correlations to proteins related to top GOCC term extrinsic component of membrane, GOCC external encapsulating structure, and GOBP protein folding. In addition to protein cluster correlations, there were 231 proteins that correlated to *APOE* genotype (148 negatively correlated, 83 positively correlated; 44 proteins shared between AD plaque and AD non-plaque; 47 shared with MCI non-plaque). The most significantly correlated protein to *APOE* genotype was SYNJ2BP (p = 6.24 × 10^− 5^; R^2^ = 0.27) with a negative correlation. There were no proteins common to all groups with a correlation to *APOE* genotype, but there were three shared proteins in MCI and AD plaque and non-plaque tissue (GAPDH, ACTN4, DTD1). There were no protein cluster correlations in AD non-plaque tissue to regional CAA score, Braak, or sex.

## Discussion

This is the first study to evaluate the MCI plaque proteome with comparison to large AD and control cohorts. We identified 135 proteins that were differentially abundant in MCI plaque tissue when compared to neighboring non-plaque tissue, 156 proteins in AD plaque tissue, 100 of these significant proteins were shared in both disease groups, and there was a moderate correlation of these protein levels across disease groups. These differentially abundant proteins were most significantly associated with increased inflammation and lysosome proteins in MCI and AD and decreased myelin proteins particularly in AD. Further, we identified differentially abundant proteins in MCI and AD non-plaque tissue when compared to control non-plaque tissue, associated with structural constituent of chromatin in MCI and negative regulation of DNA recombination and autolysosome in AD, with decreased proteins associated with actin-myosin filament in AD. Evaluating proteins further by case history identified a number of proteins that corresponded to pathology levels within each age-matched disease group, including in preclinical AD and MCI cases, which may implicate proteins involved in AD pathology progression and resistance. Further, *APOE* genotype correlated to a number of protein differences, particularly in MCI cases.

Our results suggest many similarities in the MCI and AD plaque proteomes that were related to inflammation and lysosome proteins, although with more differences in myelin proteins in AD plaque tissue. The most enriched proteins in MCI and AD were associated with the lysosome (40 shared proteins), which is consistent with our previous observations in LOAD, EOAD, and DS [19, 56]. These 40 shared lysosomal proteins included canonical lysosome protein LAMP1 enrichment in both MCI and AD plaque tissue, as well as the novel plaque protein PIP4P2. Enrichment of lysosome proteins in extracellular plaque tissue may occur as part of the pathogenesis of AD, following the inside-out amyloid hypothesis with Aβ42 intraneuronal accumulation that results in neuronal degeneration and release of Aβ42 extracellularly [23, 19, 24, 16, 66]. Many plaque enriched proteins in MCI and AD were also associated with an inflammatory response (18 shared proteins including APOE, CLU, MDK, PTN, C3, C4A, HLA-DRB1, VAMP7 among others). These proteins may contribute to synapse loss, dystrophic neurite formation, and Aβ pathology [19, 56, 50]. APOE was enriched in MCI and AD plaque tissue as well as in MCI non-plaque tissue, and it was the most significantly correlated protein to Aβ peptide levels across all samples driven particularly by MCI *APOE* E3 cases in plaque tissue. Our previous study in LOAD plaque tissue and CAA have also identified enrichment of APOE protein in these lesions [56, 43]. APOE is predominantly expressed by astrocytes as well as reactive microglia and other stressed cells, can be secreted, includes functions such as lipid transport, and binds several receptors [35]. APOE has differential binding properties by *APOE* genotype for lipids and Aβ, including increased microglial uptake of Aβ and altered inflammatory profiles in the context of *APOE* E3 when compared to *APOE* E4 [88, 35, 10, 86, 75, 71]. These *APOE* genotype associated differences are still not well understood and thus future studies will be of interest to characterize how higher APOE protein in MCI cases than in AD and control may facilitate an anti-inflammatory mechanism or other protective mechanisms that limit cognitive decline. GBA1 was a notable novel inflammatory response-related protein enriched in MCI plaques that is also associated with lysosomal function.

Midkine (MDK) was a highly enriched protein observed in both MCI and AD plaque tissue. MDK moderately correlated to Aβ peptide levels across all samples, and MDK was among the top enriched shared proteins when compared to our previous CAA analysis [43]. Previous studies have shown MDK histologically in human AD plaque tissue and CAA [50, 96]. MDK is a heparin-binding neurotrophic growth factor and also plays a role in the neuroimmune axis, with expression induced in neurons, astrocytes, and immune cells and thus is altered in various disease states [65]. Knockout of *MDK* in an AD mouse model (5XFAD) resulted in increased amyloid and microglial activation, and *in vitro* studies showed that recombinant MDK mitigated fibril formation of both Aβ40 and Aβ42 by thioflavin T fluorescence, circular dichroism, negative-stain electron microscopy, and nuclear magnetic resonance analyses [96, 63]. However, another study with differing experimental variables showed that overexpression of MDK in an AD mouse model (CRND8) increased plaque tissue, CAA, and astrocytosis [50]. Overall, these studies suggest that MDK may be protective against amyloid pathology in AD by interfering with specific stages of Aβ aggregation, but this requires further characterization.

HTRA1 was enriched in plaque tissue from both MCI and AD cases, as was one of its substrates CLU. Plaque and CAA enrichment of HTRA1 has been reported previously [31, 19, 20, 93, 56, 43, 25]. Recently HTRA1 was reported as being among the most significantly enriched proteins in the proteome of human Aβ oligomers [37]. HTRA1 is a secreted serine protease and plays a role in physiological functions associated with protein quality control with many targets that allow for disaggregation of proteins like APP/Aβ, tubulins, tau, synuclein, TDP-43, FUS [12, 25, 13, 70, 26]. When evaluating synuclein seeding, lower intracellular rather than extracellular HTRA1 levels better protected against seeding [12]. Further, HTRA1 disaggregase activity was proteolytic-independent in solubilizing synuclein. *HTRA1* knockout mice develop vascular pathology, but this model has not been evaluated in the context of AD and amyloid plaque formation yet [98, 38]. Among the multiple substrates of HTRA1, CLU is a substrate [3] and was enriched in MCI and AD plaque tissue, had a strong correlation to HTRA1 protein levels, and also was the most significantly correlated protein in MCI plaque tissue to the Aβ peptide. CLU not only is an HTRA1 substrate, but it was also among the proteins associated with an increased inflammatory response in plaque tissue. CLU is expressed primarily by astrocytes as well as microglia, plays a role in complement system activation, and data also supports that CLU plays a protective role in AD particularly via the soluble form when compared to the intracellular form [14, 11, 52, 10].

TMEFF2 was enriched in plaque tissue from both MCI and AD cases. Plaque enrichment of TMEFF2 has been reported previously in EOAD, DS, preclinical AD, and LOAD [19, 93]; as well as increased in bulk tissue of AD cases when compared to controls [36]. By proteomics TMEFF2 was decreased in the cerebrovasculature of AD cases regardless of CAA status when compared to control cases [92], was not detected in cases with CAA from other proteomic studies [43, 97, 67, 31, 34, 28, 55, 60], nor in choroid plexus [46]. TMEFF2 is a transmembrane proteoglycan with three isoforms, proteolytically cleaved and can be shed from the cell surface including by the metalloproteinase ADAM17, and it plays a role in physiological functions associated with metabolism, neuroprotection, apoptosis, embryonic development, onco-suppression, and endocrine function, with knockout mice smaller, fail to gain weight, and die at weaning age [58]. In previous AD-related studies, TMEFF2 was observed in AD plaque tissue histologically but it was not clear whether it was present in CAA [83]. Additionally, another study identified that TMEFF2 binds Aβ and is neuroprotective with *in vitro* and *in vivo* models [32]. Our immunohistochemistry results showed a better correlation of TMEFF2 and Aβ intensity levels in preclinical AD and MCI than in AD cases, with more variability in the AD group. Future studies should evaluate TMEFF2 protein expression (including isoforms) in AD cases that may support its role as neuroprotective and how other case history may influence the expression of TMEFF2.

In plaque tissue, oligodendrocyte and myelin proteins were particularly depleted in AD cases in comparison to MCI cases. In AD many myelin associated proteins were depleted in plaques (e.g. two MBP isoforms, PLP1, MAG, MOBP, CNP, MOG, PLLP, CLDN11, and BCAS1), while MOBP was significantly depleted in MCI. In our previous plaque proteome study, we also identified depleted oligodendrocyte proteins in plaque tissue in all AD subtypes examined [56]. Other studies have described alterations to myelin with aging and in AD, with damage to myelin seen in preclinical AD as well as severe AD in multiple brain regions, and in AD mouse models including with myelin defects that increased Aβ deposition and altered microglial response [17, 39, 99]. Changes to myelin in AD are also reflected in the lipidome, severe in the gray matter and also observed in the white matter, and in a mouse model is associated with neuroinflammation and cognitive deficits [73, 27, 29, 39]. SMOC1 is an oligodendrocyte progenitor cell (OPC) protein, but we detect an enrichment of this protein in MCI and AD plaque tissue, as other previous studies have observed [56, 19, 5]. SMOC1 can be secreted by OPCs and was present in higher levels of plaque tissue in temporal cortex than in frontal cortex, which may reflect the level of OPCs present [5]. The role SMOC1 plays in specific cell types in the context of AD is of interest for future studies. Non-plaque tissue did not show GO term enrichment for altered oligodendrocyte or myelin proteins, but did include decreased MBP and ADGRL3 in MCI, and decreased TBC1D5 and increased PTBP2 in AD.

In non-plaque tissue, comparison of MCI and AD to control cases identified a variety of differentially abundant proteins and there was only a mild correlation of protein levels across disease groups. Notable examples included the Aβ peptide and GFAP, enriched in both MCI and AD non-plaque tissue, which was also observed in our previous proteomic study of DS and EOAD but did not reach significance in LOAD likely associated with brain region differences that may occur with the combined temporal cortex and hippocampal regions [56]. GFAP is one of the most consistently increased proteins in human AD brain tissue [4]. Other studies have observed increased GFAP, with increased levels in bulk brain tissue by proteomics corresponding to higher amyloid, and a linear increase of GFAP histologically over the disease course in the plaque vicinity which continued after amyloid levels plateaued but did not increase beyond the plaque or NFT vicinity in temporal cortex [68, 81]. In the current dataset, increased non-plaque proteins in AD were associated with GOCC term autolysosome (LAMP1, LAMP2, SQSTM1, FTL, HLA-DRB1) and GOBP term negative regulation of DNA recombination (histone proteins H1–0, H1–2, H1–4, H1–5), which included some similarities to MCI and our previous proteomic study in LOAD [56]. Altered lysosome proteins in both plaque and non-plaque tissue are consistent with previous studies of the inside-out amyloid hypothesis as noted above. Histone proteins were enriched in AD, and to a lesser extent in MCI. Previous studies show the H1 histone protein can be increased in chronic neurodegenerative disease including outside the nucleus in neurons and astrocytes, and that H1 interacts with amyloid-like structures [8, 22]. We also identified increased H3 and H4 histone proteins, which previous studies showed increased intracellularly in AD histologically and levels correlated to inflammatory (GFAP and HLA) and AD pathology (amyloid and tau) protein levels in all layers of the inferior temporal cortex but not cerebellum [64]. These results may indicate both a compensatory response and an effect on promoting pathology. When comparing MCI and AD in non-plaque tissue, there were few shared proteins and a mild correlation of protein fold-changes that indicate more differences in the non-plaque tissue associated with disease stage.

We further evaluated protein differences that were associated with Aβ pathology levels by WGCNA. RIMS1 had a positive correlation to regional plaque score in control cases, was mildly (FC < 1.5) elevated in MCI non-plaque tissue, and was decreased in AD non-plaque tissue. *RIMS1* plays a role in calcium triggered neurotransmitter release, with distinct expression patterns in brain tissue for RIMS1 and the related RIMS2 [79, 80]. In a mouse model, *RIMS1* knockout results in severe behavioral abnormalities in spatial learning and fear conditioning [72], and redundancy was identified at some synapses including in hippocampal excitatory and inhibitory neurons where RIMS1 could compensate for RIMS2 but not vice versa [80]. RIMS2 was not differentially abundant in our study. Further, RIMS1 phosphorylation is altered in a young APP/PS1 mouse model, which is associated with NMDAR-mediated synaptic response inhibition and impaired long term potentiation (LTP) and hippocampus-dependent memory [90]. Previous proteomic studies in human AD tissue also identified decreased RIMS1 in bulk tissue [61, 4]. Similar to RIMS1, the synaptic protein SV2A linked with cognitive decline [40] was mildly increased in MCI non-plaque tissue, mildly decreased in AD non-plaque tissue, and had a positive correlation to control non-plaque tissue regional plaque score. These results are consistent with synaptic integrity loss in AD [84] and also suggest that synaptic integrity may be maintained as a result of increased RIMS1 in the non-plaque tissue of these preclinical AD and MCI cases, potentially related to protective factors that limit pathology and cognitive symptom development and/or a compensatory response that results in resistance or resilience in developing pathology.

We also evaluated protein differences that were associated with *APOE* genotype by WGCNA. The most significant cluster among all WGCNA analyses was identified in MCI plaque tissue with a negative correlation to *APOE* genotype. This cluster had top associations with GOCC extracellular exosome and GOBP pyridine-containing metabolic process, indicating that this cluster of proteins had higher abundance levels in APOE E2 cases and lower abundance in *APOE* E3 and E4 cases. The cluster included GAPDH, which was elevated in *APOE* E2 cases in MCI and AD non-plaque tissue as well as plaque tissue (not in control non-plaque). GAPDH has multiple functions including in glycolysis, and it has been previously linked with AD including binding and modifying Aβ pathology [77]. However, there have been no studies evaluating how *APOE* genotype may alter GAPDH function in the context of AD. In addition to protein cluster correlations, there were a number of proteins that correlated to *APOE* genotype, with the most significant in MCI plaque tissue being DNAJA1 with a negative correlation that was also seen in MCI non-plaque tissue to a lesser extent. DNAJA1 had higher protein abundance in *APOE* E2 cases and less in *APOE* E3 and E4 cases. Previous AD proteomic studies identified increased DNAJA1 in bulk brain tissue when compared to control cases in frontal cortex, sensory cortex, and hippocampus [100, 94, 36], and decreased in AD as measured by western blot in hippocampus and temporal cortex [76, 1]. DNAJA1 binds Aβ42 and facilitates aggregation into small oligomers with translocation to mitochondria in yeast and *Drosophila melanogaster* AD models, which may facilitate mitochondria-dependent Aβ42 degradation unless oversaturated by amyloid [76, 85]. Over-expression of DNAJA1 facilitates tau clearance and knockdown results in tau accumulation in a cell culture model [1, 2]. As noted above, further studies are needed to characterize how *APOE* genotype influences AD pathology, including in the context of other genetic and extrinsic factors, as 34% of our age-matched control group included *APOE* E4 carriers.

Our study had some limitations. Our proteomics approach is less sensitive in detecting membrane proteins, insoluble proteins, and low abundance proteins. Clinical variables warrant further evaluation in future studies, including age and disease duration to better understand proteins associated with resistance and resilience of developing AD pathology and cognitive symptoms, as well as associations with intrinsic genetic and extrinsic factors [59] that may influence heterogeneity in AD.

In conclusion, we conducted the most extensive proteomic analysis of microdissected plaque proteomes in MCI and AD to date. Our results provide insights into MCI and AD molecular mechanisms, novel biomarkers, and potential novel therapeutic targets.

## Supplementary Material

Supplementary Files

This is a list of supplementary files associated with this preprint. Click to download.

- PlaqueproteomicsSupplementaryTables113011626.xlsx
- MCIADPlaqueProteomicssupplement030226DL.docx

## Figures and Tables

**Table 1:** Case History Summary

Group	# Cases	Mean Age at Death (years)	Sex	Mean PMI (hours)	*APOE* Genotype	Regional Temporal Cortex Plaques (Score 0–5)	Braak Stage	ABCA Score	ABCB Score	ABCC Score
Control	62	86.6 ± 7.0	37 F/25 M	12 ± 13	17 E2; 24 E3; 21 E4	1.0 ±1.2	2.2 ± 1.1	0.8 ± 0.7	1.3 ± 0.6	0.5 ± 0.7
MCI	36	89.3 ± 5.8	25 F/11 M	9 ± 6	9 E2; 11 E3; 16 E4	3.0 ±1.4	3.5 ± 0.9	2.1 ± 0.5	1.9 ± 0.4	2.1 ± 0.6
AD	53	90.2 ± 6.3	40 F/13 M	11 ± 9	6 E2; 24 E3; 23 E4	4.0 ±1.0	5.1 ± 0.3	3.0 ± 0.0	3.0 ± 0.0	3.0 ± 0.0

*APOE* E2 carriers: all E2/E3 heterozygous, except 1 MCI E2/E2

*APOE* E3 carriers: all E3/E3 homozygous

*APOE* E4 carriers: all E3/E4 heterozygous, except 1 control, 1 MCI, 5 AD E4/E4

## Data Availability

All data are available in the supplementary data and the mass spectrometry data in the online repository at the MassIVE repository (https://massive.ucsd.edu/) under accession MSV000100660.
